# Drug discovery and preclinical testing of drug candidates for developmental and epileptic encephalopathies

**DOI:** 10.1111/epi.18581

**Published:** 2025-08-06

**Authors:** Heidrun Potschka, Daniel Pérez‐Pérez

**Affiliations:** ^1^ Institute of Pharmacology, Toxicology, and Pharmacy Ludwig‐Maximilians‐Universität (LMU) of Munich Munich Germany

**Keywords:** antiseizure medication, cannabidiol, fenfluramine, orphan drug, preclinical drug development

## Abstract

Drug development for developmental and epileptic encephalopathies (DEEs) follows different strategies on one hand including disease‐targeting precision medicine approaches considering the genetic variants and pathomechanisms in DEEs and on the other hand including therapeutic approaches with novel targets or second‐generation drug candidates that may be of interest beyond selected DEEs. Although the first group of approaches can only be tested in dedicated DEE models, assessment in induced non‐specific seizure and epilepsy models may provide valuable information if the mechanism of action implies a broader spectrum of efficacy. Data from such models can inform about general anti‐seizure efficacy, efficacy against different seizure types including a possible broad‐spectrum potential, dose range, and “therapeutic” plasma/brain concentrations. However, only dedicated DEE models will guide selection of the best candidates with a favorable efficacy and tolerability spectrum for specific DEEs. Several DEE models have already been used for preclinical testing of therapeutic approaches. Testing in these specific models can provide information about the effects on seizure generation; spread of seizure activity; epilepsy development; survival; behavioral, cognitive, and motor function; and about tolerability. On the other hand, we still face several limitations and challenges including lack of models for many DEEs, incomplete penetrance of the phenotype, high mortality, low throughput, limited knowledge concerning pharmacology and predictive validity, and species differences in development and disease course. In this review, we provide an overview of the preclinical efficacy data of approved orphan drugs in both model types and discuss the current state‐of‐knowledge concerning predictive validity. In conclusion, testing strategies need to be carefully tailored to the candidate drug or therapeutic approach. In this context, there is an urgent need for development of further specific DEE models and for a comprehensive characterization of the face and predictive validity of existing and future DEE models.

**Key points**

- Drug development strategies must consider the complexity and severity of developmental and epileptic encephalopathies (DEEs).
- Therapeutic approaches for DEEs are divided into those not necessarily specific for DEEs and precision medicine approaches.
- Antiseizure medications (ASMs) approved for DEEs showed inconsistent efficacy profiles in non‐specific seizure and epilepsy models.
- ASM testing in DEE models can provide insights into seizure‐ and non–seizure‐related outcomes relevant for translation of the findings.
- There is a need to intensify our efforts to further develop and to comprehensively characterize genetic models of different DEEs.

## 1 INTRODUCTION

### 1.1 Drug development concepts for developmental and epileptic encephalopathies

Progress in technologies, data management, and data science allows accelerated detection of genetic variants causative of epilepsy, identification of the functional consequences, characterization of syndromic phenotypes, and definition of syndromes including developmental and epileptic encephalopathies (DEEs).

Drug development strategies must consider that DEEs represent a heterogeneous group of epilepsy syndromes with early onset, severe epileptic seizures, and developmental impairment.^1^, ^2^ The full syndromic spectrum is often characterized by different seizure types; various non‐seizure symptoms with pathophysiological mechanisms that can affect cognition; mood, behavior, and motor function; and a high risk of sudden unexpected death in epilepsy (SUDEP).^3^

**FIGURE 1:**
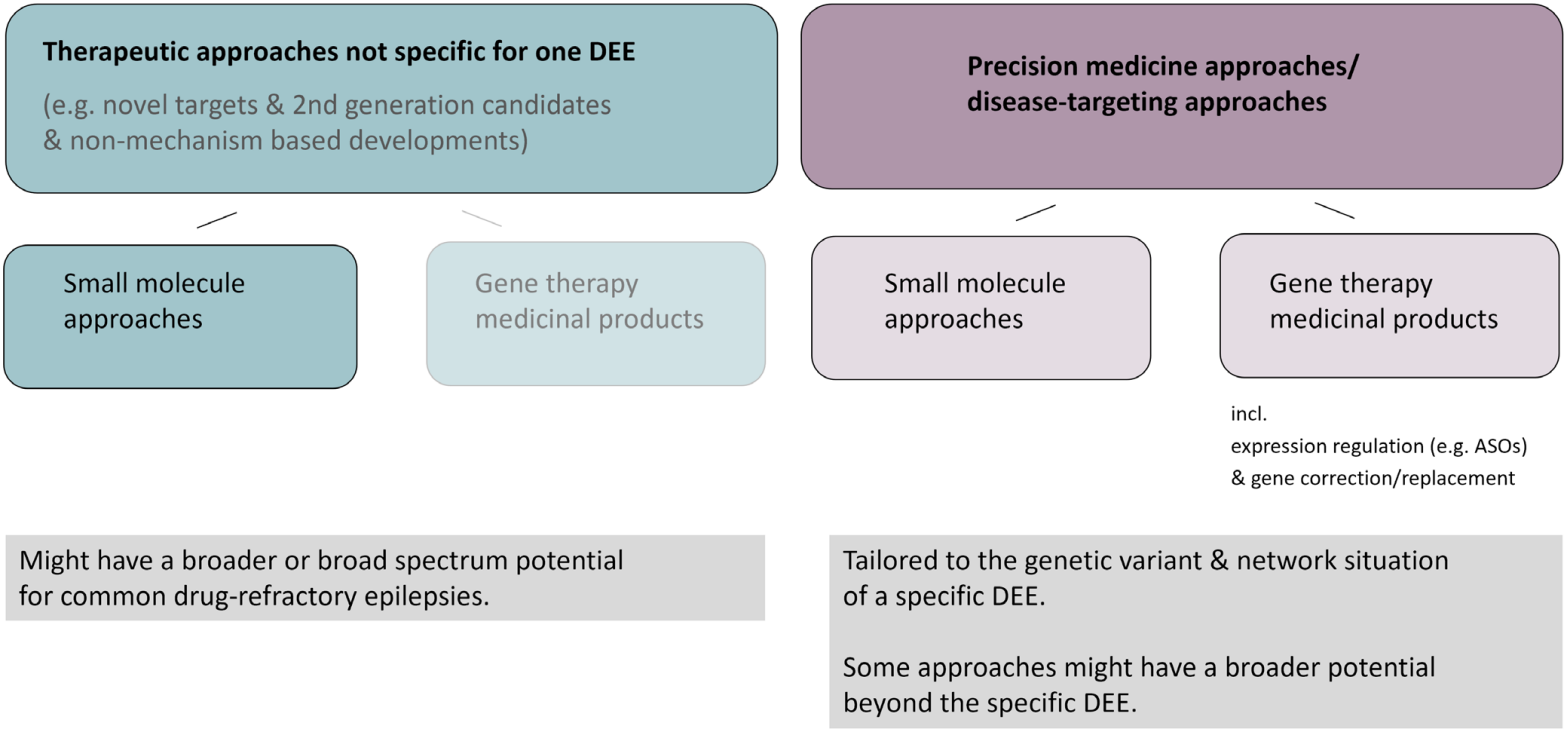
Categorization of drug development strategies for developmental and epileptic encephalopathies (DEEs). The boxes in the bottom row summarize the characteristics with regard to the potential for further indications from narrow to broad. ASOs, antisense oligonucleotides.

Therapeutic approaches under development can be broadly divided into therapeutic approaches that are not necessarily specific for one or selected DEEs on the one hand and precision medicine or disease targeting approaches on the other (Figure 1).

The first group may have a broader or broad‐spectrum potential that could extend to various DEEs and to common drug‐refractory epilepsies. Corresponding approaches can be based on second‐generation candidates aiming for an optimized targeting of known or novel target sites. In general, these approaches are based predominantly on traditional small molecule drug candidates. Examples in the drug development pipeline for DEEs include novel drug candidates that aim to more specifically target serotonergic neurotransmission considered second generation to fenfluramine.^4^

The hope for disease‐targeting and possibly disease‐modifying therapies relies on the development of precision medicine approaches that are tailored to the genetic variant and associated network situation of a particular DEE.^3^, ^5^ Respective strategies can be based on small molecule approaches, for example, aiming for selective targeting of voltage‐gated ion channels such as specific sodium, calcium, or potassium channel subtypes or ionotropic receptors, which are directly or indirectly linked to the pathophysiological consequences of the genetic variant of one or more selected DEEs (e.g., see Guerrini et al.^3^ and Bialer et al.^6^).

Alternate promising developments are based on *advanced therapy medicinal products* (ATMPs) and, in particular, the subgroup of *gene therapy medicinal products* (GTMPs; for definition proposed by the European Federation of Pharmaceutical Industries and Associations [EFPIA] see https://www.efpia.eu/media/zrwhbk1w/efpia‐position‐paper‐on‐gtmp‐definition_docx.pdf) comprising various approaches, which aim to generate a therapeutic effect through a modulation of gene expression rates or by replacing genes.^3^, ^5^ Strategies targeting expression rates are for instance in development for DEEs with haploinsufficiencies such as Dravet syndrome. The approaches that aim to increase the sodium voltage‐gated channel alpha subunit 1 protein (Na_v_1.1) in patients with Dravet syndrome and that have entered clinical studies comprise a viral vector–based therapy with expression of a transcription factor as well as an antisense oligonucleotide‐based approach targeting an alternate splicing event (https://clinicaltrials.gov/study/NCT04740476).^7^, ^8^

In this critical review, we provide an overview of preclinical studies that have assessed antiseizure medications (ASMs) already licensed as orphan drugs for DEEs. Based on these data sets we discuss the limitations of these models, the challenges associated with their use, and the current state‐of‐knowledge concerning their informative value and predictive validity.

To identify relevant publications in the PubMed data base, we applied a semi‐systematic approach using the search strings provided in Supporting Information.

## 2 TESTING IN NON‐SPECIFIC SEIZURE AND EPILEPSY MODELS

**FIGURE 2:**
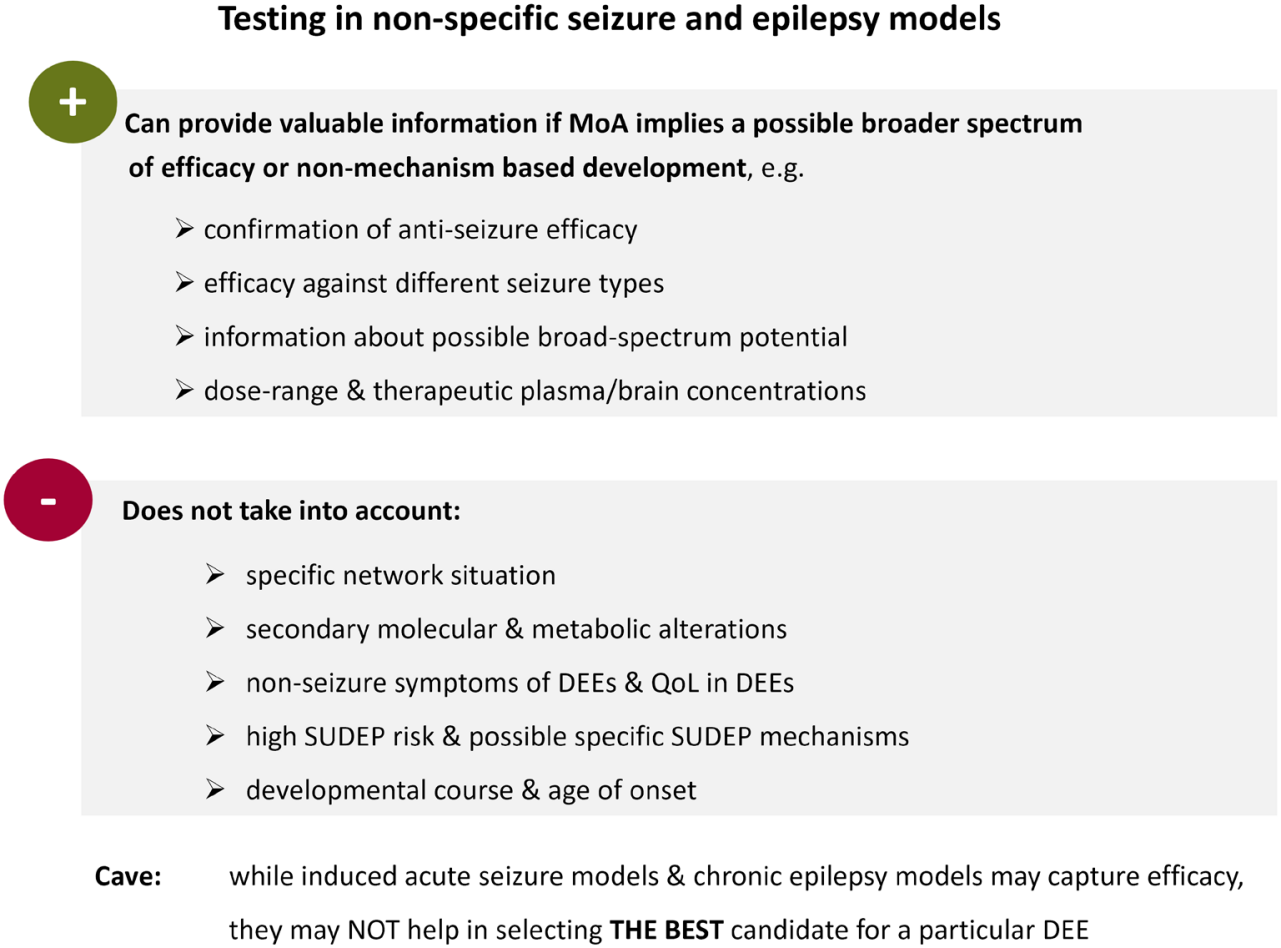
Opportunities and limitations for testing drug candidates for developmental and epileptic encephalopathies (DEEs) in non‐specific seizure and epilepsy models. MoA, mechanism of action; QoL, quality of life; SUDEP, sudden unexpected death in epilepsy.

If the mechanism of action (MoA) of a drug candidate does not exclude a broader spectrum of efficacy, assessment in non‐specific induced models of seizures and epilepsy can provide valuable information (Figure 2). For key information about the technical setup and execution of common non‐specific seizure and epilepsy models, see Supporting Information.

Data from such models can confirm general antiseizure efficacy. Models with induced seizures can allow determination of effects on seizure thresholds and ictogenesis, seizure spread, and seizure termination. Assessment in models with a different predictive validity can provide information about efficacy against specific seizure types (Table 1),^9^ which can be of relevance for patients with DEEs with a mix of different seizure types. Moreover, testing of drug candidates in non‐specific seizure and epilepsy models can inform about a possible broad‐spectrum potential in different etiologies and associated epilepsy types,^9^ which could be important concerning an expansion of the indication areas in the future. An expansion from one selected DEE indication to other DEEs has already taken place and might go even further with fenfluramine and cannabidiol.

**TABLE 1:** Models of seizures and epilepsy frequently used in pre‐clinical research.

Type of model	Model	Mode of induction	Type of seizure/epilepsy modeled
Acute	Electrically induced seizures	Electrically induced generalized tonic–clonic or psychomotor seizures in animals	Generalized tonic–clonic seizures (MES/MEST) Focal‐onset seizures (6 Hz) Drug‐resistant seizures (6 Hz 44 mA)
	Chemically induced seizures	Systemic administration of convulsant drugs to induce self‐limited seizure activity	scPTZ and ivPTZ: suggested predictive validity for non‐motor (absence) and myoclonic seizures—but: false positives and negatives reported
Chronic	Kindling model (KDL)	Repeated electrical or chemical stimulation that triggers seizures with increasing severity and persistent reduction in seizure thresholds	Focal and generalized seizures Temporal lobe epilepsy (aKDL) Drug‐resistant epilepsy (e.g., lamotrigine‐resistant aKDL or cKDL)
	Post‐SE induced epilepsy	Systemic or intracerebral administration of convulsant drugs induce status epilepticus and, after a latency period, SRS	Focal‐onset seizures Temporal lobe epilepsy Drug‐resistant temporal lobe epilepsy (IHK)
	Post‐traumatic epilepsy	Induction of a controlled mechanic brain injury induces epilepsy after a latency period	Post‐traumatic epilepsy
Specific models	Genetically selected strains	Genetic selection of animals with spontaneous generalized non‐motor (absence) seizures or with increased susceptibility to induced seizures	Epilepsy with generalized non‐motor (absence) seizures (GAERS and WAG/Rij) Audiogenic seizures (DBA/1, DBA/2, GEPR3, WAR, and AGS)
	Infection‐induced epilepsy	Intracerebral administration of TMEV induces acute seizures and long‐lasting increased seizure susceptibility in subgroups of animals	Infection‐associated seizures and epilepsy
	Genetically modified animals	Induction of genetic modification in animals to simulate the human epilepsy‐related genetic alterations	Wide spectrum depending on the mutation (genetic epilepsies and DEEs). Acute seizures can be induced by different methods.
In vitro	In vitro brain slices	In vitro evaluation of brain slices with spontaneous (obtained from animals with spontaneous recurrent seizures) or induced (chemically or electrically) epileptiform activity	Chemically induced epileptiform activity. Spontaneous epileptiform activity (slices derived from rats with SRS).

*Note*: This table considered information from Löscher and Klein^9^ and Barker‐Haliski and White.^10^

Abbreviations: AGS, audiogenic seizure susceptible rats; aKDL, amygdala KDL; cKDL, corneal KDL; DBA, Dilute Brown Non‐Agouti; DEEs, developmental epileptic encephalopathies; GAERS, Genetic Absence Epilepsy Rats from Strasbourg; GEPR, Genetically Epilepsy‐Prone Rats; Hz, Hertz; IHK, Intrahippocampal Kainic acid mouse model; ivPTZ, intravenous pentylenetetrazole; mA, milliAmps; MES, maximal electroshock seizure; MEST, MES threshold; scPTZ, subcutaneous pentylenetetrazole; SE, status epilepticus; SRS, spontaneous recurrent seizures; TMEV, Theiler's murine encephalomyelitis virus; WAG/Rij, Wistar‐Albino‐Glaxo from Rijswijk rats; WAR, Wistar Audiogenic Rat.

Such a potential needs to be considered early in the context of strategic decision‐making processes when it comes to either applying for an orphan drug designation or developing a drug for a broader indication area such as epilepsy with drug‐refractory focal‐onset seizures. Finally, testing in less time‐consuming non‐specific models such as acute seizure models can in general inform about pretreatment times, efficacious and tolerated doses, and “therapeutic” plasma/brain concentrations, thereby guiding the selection of dose ranges for further testing in more elaborate genetic DEE models and for toxicology and safety studies.

In this context it needs to be critically considered that induced acute seizure models and chronic epilepsy models can capture the efficacy of drug candidates suitable for seizure reduction or control in DEEs, but they do not necessarily help to select the best candidates for a particular DEE (Figure 2). Even worse, it should be noted that drugs selected in “standard” screening programs might not only lack efficacy but can also exert detrimental effects. One prominent example is the contraindication of traditional modulators of voltage‐gated sodium channels in patients with Dravet syndrome.^11^ In the specific network situation characterizing Dravet syndrome, the consequences of the sodium voltage‐gated channel alpha subunit 1 (*SCN1A*) genetic variants and the resulting interneuronopathy can be worsened by non‐selective modulators of voltage‐gated sodium channels.^12^, ^13^, ^14^ As this class of ASMs has been identified and validated by traditional induced seizure and epilepsy models,^9^, ^15^ this example underscores the need for additional testing in genetic DEE models, thereby assessing efficacy and tolerability in models, which should better recapitulate the specific network situation in a particular DEE. It is therefore emphasized that induced acute seizure models and chronic epilepsy models should never be used as a filter in the sense of go and no‐go decision‐making when identifying or selecting drug candidates for DEEs.

Considering the potential and the limitations of non‐specific seizure and epilepsy models (Figure 2), it is of particular interest to review available efficacy data for orphan drugs that have been approved for selected DEEs. Retrospective analysis of preclinical datasets for approved orphan drugs can inform about the informative value and predictive validity of seizure and epilepsy models. Various orphan drugs licensed for specific DEEs (Table 2) have indeed been evaluated in non‐specific models (Table 3 and Tables S2–S7).

**TABLE 2:** Summary information on the orphan drug designation of different antiseizure medications in developmental and epileptic encephalopathies.

Antiseizure medication	Indication(s)	Year of marketing authorization		Orphan drug designation(s)	Mechanism(s) of action^a^
		EMA	FDA		
Fenfluramine	Dravet syndrome	2020	2020	*CDKL5* deficiency disorder	Enhancer of serotonergic neurotransmission (direct and indirect agonist) Modulator of Sigma‐1^16^
	Lennox–Gastaut syndrome	2020	2022		
Stiripentol	Dravet syndrome	2007	2018		Positive allosteric modulator of GABA_A_ receptors Lactate dehydrogenase inhibition^17^ Modulation of voltage‐gated sodium and T‐type calcium channels
Ganaxolone	*CKDL5* deficiency disorder	2023	2022	Tuberous sclerosis complex	Positive allosteric modulator of GABA_A_ receptors
Everolimus	Tuberous sclerosis complex	2010	2011		mTOR inhibitor
Rufinamide	Lennox–Gastaut syndrome	2007	2008		Modulator of voltage‐gated sodium channels
Cannabidiol	Dravet syndrome	2019	2018		GPR55 antagonist TRPV1 agonist Adenosine reuptake inhibitor (= inhibitor of the ENT1 transporter)
	Lennox–Gastaut syndrome	2019	2018		
	Tuberous sclerosis complex	2019	2020		

*Note*: Information from: https://www.ema.europa.eu/en/homepage, https://www.fda.gov/ and https://www.orpha.net/.

Abbreviations: CDKL5, Cyclin‐dependent kinase like 5; EMA, European Medicines Agency; ENT1, equilibrative nucleoside transporter 1; FDA, U.S. Food and Drug Administration; GABA, γ‐aminobutyric acid; GPR55, orphan G protein‐coupled receptor‐55; mTOR, mammalian target of rapamycin; TRPV1, Transient receptor potential vanilloid‐1.

^a^ Please note that the evidence level for the relevance of the mechanisms largely differs.

**TABLE 3:** Literature information: Efficacy of approved orphan drugs in non‐specific seizure and epilepsy models.

Model	Species	Antiseizure medications: Line 1—route of administration: direction of the effect (median effective dose [mg/kg]; protective index); Line 2—comments/additional information					
		Fenfluramine	Stiripentol	Ganaxolone	Everolimus^b^	Rufinamide	Cannabidiol
Maximal Electro‐shock Seizure (MES) test	Rats	i.p.: ↓ (10.7; 2.6^&^)^18^, ^19^	i.p.: ↓ (240; ‐)^20^	p.o.: ↓ (58.4; 0.8)^21^		i.p.: ↓ (7; >50^&^)^22^	i.p.: ↓ (‐; ‐)^23^
		‐	Temporal electrode	‐		‐	Neonatal/young
						p.o.: ↓ (6.1; 163^&^)^24^	i.p.: ↓ (68.8; ‐)^25^
						‐	Adult
							i.p.: ↓ (88.9; 5.6^&^)^26^
							Adult
							i.p.: ↓ (50; ‐)^27^
							Adult
							i.p.: ↓ (53.2; 9.4)^28^
							‐
	Mice	i.p.: ↓ (8.1; 5.5)^19^, ^29^	i.p.: ↓ (277.7; ‐)^30^, ^31^, ^32^	i.p.: ↓ (29.7; 1.1)^21^	p.o.: ≈ (‐; ‐)	i.p.: ↓ (15.5; >32.2)^24^	i.p.: ↓ (190; ‐)^33^
		‐	‐	‐	‐	‐	‐
		i.p.: ≈ (‐; ‐)^18^				p.o.: ↓ (23.9; >41.9)^24^, ^34^	i.p.: ↓ (41.9; ‐)^35^
		‐				‐	‐
		i.p.: ↓ (2.9; ‐)^36^					i.p.: ↓ (83.5; 5.1)^26^
		‐					‐
							i.p.: ↓ (118; 1.5^%^)^37^, ^38^
							‐
							i.p.: ↓ (120; 1.5^%^)^27^
							‐
							i.p.: ↓ (80; 3.4)^28^, ^39^
							‐
							i.c.v.: ↓ (26; ‐)^40^
							ng/mouse
Pentylene‐tetrazole (PTZ) induced seizures	Rats	i.p.: ↓ (‐; ‐)^41^	i.p.: ↓ (‐; ‐)^42^	i.p.: ↓ (7.8; 1.8)^21^, ^43^		p.o.: ≈ (‐; ‐)^24^	i.p.: ↓ (‐; ‐)^23^
		s.c. PTZ	i.p. PTZ low dose	s.c. PTZ		‐	s.c. PTZ (neonatal/young)
		i.p.: ≈ (‐; ‐)^18^	i.p.: ↓ (‐; ‐)^44^	p.o.: ↓ (21; 2.3)^21^			i.p.: ↓ (‐; ‐)^45^
		s.c. PTZ	s.c. PTZ	s.c. PTZ			s.c. PTZ
				s.c.: ↓ (3.5; 1.62)^46^, ^47^			i.p.: ↓ (‐; ‐)^48^, ^49^, ^50^, ^51^
				s.c. PTZ			i.p. PTZ
	Mice	i.p.: ≈ (‐; ‐)^18^	i.p.: ↓ (221.3; 2.9^@^)^20^, ^52^	i.p.: ↓ (4.3; 7.8)^21^		i.p.: ↓ (54; >9.3)^24^	i.p.: ↓ (‐; ‐)^53^, ^54^, ^55^
		s.c. PTZ	i.p. PTZ	s.c. PTZ		s.c. PTZ	i.p. PTZ
			i.p.: ↓ (‐; ‐)^32^	s.c.: ↓ (‐; ‐)^56^		i.p.: ↓ (‐; ‐)^57^	‐: ≈ (‐; ‐)^58^
			s.c. PTZ	i.p. PTZ		i.p. PTZ	i.v. PTZ
						p.o.: ↓ (45.8; >21.9)^24^	i.p.: ↓ (159; 3.1)^26^, ^27^, ^55^
						s.c. PTZ	s.c. PTZ
							i.p.: ↓ (120; 2.3)^28^
							s.c. PTZ
						p.o.: ↓ (‐; ‐)^34^	‐: ↓ (‐; ‐)^55^
						i.p. PTZ	i.v. PTZ
	Zebrafish	i.p.: ↓ (‐; ‐)^59^					Bath: ↓ (‐; ‐)^60^, ^61^, ^62^
		i.p. PTZ					Bath PTZ
6 Hz induced seizures	Rats					i.p.: ↓ (42.7; 8.2^&^)^22^	
						60 V	
						i.p.: ↓ (‐; ‐)^22^	
						80 V	
	Mice	i.p.: ↓ (‐; ‐)^63^		s.c.: ↓ (1.46; ‐)^64^	p.o.: ≈ (‐; ‐)	i.p.: ↓ (23.1; >2)^22^	i.p.: ↓ (53.6; >7)^65^
		22 mA		Male‐38 mA	32 mA	32 mA	38 mA
		i.p.: ≈ (‐; ‐)^18^		s.c.: ↓ (2.9; ‐)^66^		i.p.: ↓ (32.9; >1.4)^22^	i.p.: ↓ (49.6; ‐)^35^
		32/44 mA		Male‐32 mA		44 mA	44 mA
		i.p.: ↓ (47; 0.7)^36^		s.c.: ↓ (1.5; ‐)^66^			i.p.: ↓ (164; 2.6)^26^
		44 mA		Female‐32 mA			44 mA
				i.p.: ↓ (6.3; ‐)^67^			p.o.: ≈ (‐; ‐)^68^
				32 mA			44 mA
							i.p.: ↓ (144; 1.9)^28^, ^69^, ^70^
							32 mA
							i.p.: ↓ (173; 1.6)^28^
							44 mA
Seizures in susceptible strains	Rats		i.p.: ↓ (‐; ‐)^42^	i.c.: ↓ (‐; ‐)^71^			i.p.: ↓ (‐; ‐)^72^
			WAG/Rij	WAG/Rij			Audiogenic/GEPR‐3
							i.p.: ↓ (82.4; 5.5)^73^
							Audiogenic/AGS
							i.v.: ↓ (14.9; 2.1)^73^
							Audiogenic/AGS
							p.o.: ↓ (17; >5.8)^74^
							Audiogenic/AGS
							p.o.: ↓ (12; >8.3)^74^
							MES/AGS
							i.p.: ↓ (‐; ‐)^75^
							SWD/GAERS
	Mice	i.p.: ↓ (11.8; ‐)^29^					
		Audiogenic/DBA/2					
		i.p.: ↓ (21; ‐)^76^, ^77^					
		Audiogenic/DBA/1					
	Other	i.p.: ≈ (‐; ‐)^78^					i.p.: ≈ (‐; ‐)^79^
		Air pressure/Gerbils					Audiogenic/GASH Hamster
*Status Epilepticus* (SE)	Rats		i.p.: ↓ (100; ‐)^44^, ^80^, ^81^	i.v.: ↓ (‐; ‐)^82^		i.p.: ≈ (‐; ‐)^83^	i.p.: ↓ (‐; ‐)^28^, ^84^, ^85^, ^86^
			Young	‐		+ MDZ and KET	‐
			i.p.: ≈ (‐; ‐)^44^				p.o.: ↓ (‐; ‐)^87^, ^88^
			Adult				‐
			i.p.: ↓ (377.6; ‐)^80^, ^81^				i.c.: ↓ (‐; ‐)^85^
			Adult				‐
Drug‐Resistant SE^a^	Rats		i.p.: ↓ (377; ‐)^80^, ^81^	i.v.: ↓ (‐; ‐)^82^			
			Young	‐			
			i.p.: ↓ (397.2; ‐)^80^				
			Adult				
	Mice			i.m.: ↓ (‐; ‐)^89^			
				‐			
Kindling (KDL)	Rats			i.p.: ↓ (4.5; 3.2)^21^	p.o.: ≈ (‐; ‐)	i.p.: ≈ (>40; ‐)^90^	p.o.: ≈ (‐; ‐)^91^
				Corneal KDL	Corneal KDL	LTG‐resistant	i.p. PTZ KDL
							p.o: ≈ (‐; ‐)^91^
							i.p. PTZ/progression
							‐: ↓ (‐; ‐)^92^
							i.p. PTZ/progression
							i.p.: ↓ (‐; ‐)^72^, ^93^
							Audiogenic/GEPR‐3 and WAR
							i.p.: ↓ (‐; ‐)^94^
							Hippocampal KDL
							i.p.: ↓ (‐; ‐)^72^, ^93^
							Audiogenic/progression/GEPR‐3 and WAR
							i.p.: ≈ (‐; ‐)^26^
							LTG‐resistant

	Mice	i.p.: ↓ (‐; ‐)^95^		s.c.: ↓ (‐; ‐)^64^, ^66^		i.p.: ↓ (‐; ‐)^96^	i.p.: ↓ (119; 4.2)^26^
		Amygdala KDL		Hippocampal KDL		i.p. PTZ/progression	Corneal KDL
		i.p.: ↓ (‐; ‐)^36^		s.c.: ↓ (6.6; ‐)^97^			i.p.: ↓ (‐; ‐)^98^
		Corneal KDL		Amygdala KDL			Hippocampal KDL/progression
				s.c.: ↓ (3.22; ‐)^99^			i.p.: ↓ (52; ‐)^98^
				i.p. PTZ KDL			Hippocampal KDL
				s.c.: ↓ (‐; ‐)^56^, ^99^			p.o.: ↓ (50; ‐)^98^
				i.p. PTZ/progression			Hippocampal KDL
				i.p.: ↓ (‐; ‐)^100^			i.p.: ≈ (‐; ‐)^55^
				s.c. PTZ/progression			i.p. PTZ/progression
							i.p.: ↓ (115; 2.4)^28^
						Corneal KDL
Post‐SE induced epilepsy	Rats				p.o.: ≈ (‐; ‐)^101^		s.c.: ↓ (‐; ‐)^102^
					Preventive		‐
					p.o.: ≈ (‐; ‐)^101^, ^103^		i.c.v.: ↓ (‐; ‐)^104^
					Therapeutic		Preventive
							i.c.v.: ↓ (‐; ‐)^104^
							Therapeutic
							p.o.: ↓ (‐; ‐)^28^
							Therapeutic
	Mice	i.p.: ↓ (‐; ‐)^95^	i.p.: ↓ (‐; ‐)^105^		p.o.: ↓ (‐; ‐)		
		IHK model	IHK model		Single		
					i.p.: ≈ (‐; ‐)^106^, ^107^		
					Sub‐/chronic		
					p.o.: ↓ (‐; ‐)^108^		
					MEST		
In vitro epileptiform activity		Bath: ↓ (‐; ‐)^109^	Bath: ↓ (‐; ‐)^110^		Bath: ≈ (‐; ‐)	Bath: ↓ (‐; ‐)^111^	Bath: ↓ (‐; ‐)^51^, ^112^
		Mg^2+^‐free induced EA	Spontaneous EA		Spontaneous EA	4‐AP induced EA	4‐AP induced EA
							Bath: ↓ (‐; ‐)^51^
							Mg^2+^‐free induced EA
							Bath: ≈ (‐; ‐)^113^
							Oxotremorine‐M induced EA

*Note*: The median toxic dose was calculated based on the rotarod performance in most of the studies. For the rest, the median toxic dose was calculated using the walk bar test (%), visual inspection (&), or the chimney test (@). The direction of the effect is illustrated by: ↓ protective effect of the compound; ≈ no evidence for a protective effect; ‐ lack of information or further explanation/comments provided.

Abbreviations: 4‐AP, 4‐aminopyridine; EA, epileptiform activity; i.c., intracerebral; i.c.v., intracerebroventricular; i.m., intramuscular; i.p., intraperitoneally; i.v., intravenous; IHK, intrahippocampal kainate mouse model; KDL, Kindling; KET, ketamine; LTG, lamotrigine; MDZ, midazolam; MEST, MES Threshold; p.o., per os; s.c., subcutaneously.

^a^ Defined by the duration of the SE before the administration of the ASM.

^b^ Efficacy information without reference was retrieved from https://panache.ninds.nih.gov/Compound.

### 2.1 Fenfluramine

First preclinical data reporting an antiseizure effect of d‐fenfluramine date back to 1983.^41^ The authors described a reduction of animals exhibiting tonic seizure activity and a complete prevention of mortality in a subcutaneous pentylenetetrazole (PTZ) model in rats in response to d‐fenfluramine (5 mg/kg intraperitoneally [i.p.], 30 min pre‐treatment time [PTT]). Another early preclinical study failed to confirm the efficacy of racemic fenfluramine in Mongolian gerbils with air‐pressure–triggered seizures and in the maximum electroshock seizure (MES) test but demonstrated a threshold‐increasing effect in the maximum electroshock seizure threshold test (MEST).^78^

Based on promising clinical data in small groups of patients, a decision was taken to develop racemic fenfluramine for therapeutic management of drug‐refractory seizures in patients with Dravet syndrome. Following confirmation of clinical efficacy, a contract research organization has assessed racemic fenfluramine in direct comparison with second‐generation serotonergic drugs, that is, selective 5‐hydroxytryptamine receptor (5‐HT(2C)) agonists in different acute mouse seizure models including the subcutaneous and intravenous PTZ model, the MES test, the MEST test, and the 6‐Hz model with stimulation at 32 and 44 mA.^18^ In this study series, fenfluramine (5–10 mg/kg, PTT 45 min) failed to exert relevant antiseizure effects in the majority of models except for limited effects observed in the MEST in mice and MES in rats. Of interest, Martin et al.^36^ reported pronounced effects of racemic fenfluramine in the MES test in mice when testing with longer PTTs of 4 h. Although these authors did not confirm antiseizure effects at doses without motor impairment in the 6‐Hz test (44 mA), Wong and colleagues^63^ observed protective effects of fenfluramine when applying a lower stimulation current (mouse 6‐Hz 22 mA model).

Several studies used acute seizure models in rodents and zebrafish to explore the MoA of fenfluramine and to compare the efficacy and MoA of its active metabolite and enantiomers. In an audiogenic seizure mouse model (Dilute Brown Non‐Agouti (DBA)/1 mouse model), Tupal and Faingold^76^ observed anti‐seizure effects of fenfluramine along with a potential to prevent seizure‐induced respiratory arrest (S‐IRA), which were counteracted by a 5‐HT4 receptor antagonist in a follow‐up study.^77^ Testing in a PTZ zebrafish seizure model revealed a protective effect that was associated with an increase in γ‐aminobutyric acid (GABA) concentrations.^59^

More recently Erenburg and colleagues^19^ compared the efficacy of the enantiomers of fenfluramine and its metabolite norfenfluramine in the MES test in rats and mice, and the 6‐Hz (44 mA) test in mice. Although all compounds failed to elicit relevant effects in the 6‐Hz assay, racemic fenfluramine and norfenfluramine and their enantiomers exerted relevant effects in the mouse and rat MES test. In rats, protective indices (median toxic dose [TD_50_]/median effective dose [ED_50_]) of the compounds ranged between 1.2 and 3.2., i.e. depending on the compound antiseizure effects were already observed at tolerated doses.^19^ As an exception, d‐norfenfluramine was associated with dose‐limiting neurotoxicity in rats, which made it impossible to determine the ED_50_.

Considering the predictive validity of the acute seizure models used, the datasets indicate an efficacy of fenfluramine against generalized motor seizures (MES, MEST) and against generalized non‐motor seizures and myoclonic seizures (PTZ).^9^ However, conclusions concerning the predictive validity of these models have been challenged repeatedly by false positives and false negatives with drugs for which preclinical data did not translate in clinical efficacy against the respective seizure types.^114^

In contrast to acute seizure models, chronic models with repeated seizure induction or with spontaneous recurrent seizures better recapitulate molecular, cellular, and network characteristics of human epilepsy.^114^ Although testing in a chronic amygdala‐kindling paradigm revealed only very limited effects of racemic fenfluramine on seizure induction in fully kindled mice, acute administration of 10 mg/kg (intraperitoneally) fenfluramine in an intrahippocampal kainate mouse model exerted relevant effects on frequent electrographic seizure events.^95^ Earlier data from a corneal kindling paradigm were rather in line with the findings from the amygdala‐kindling paradigm, as only minimal antiseizure effects were detected.^36^

Taken together the datasets collected from non‐specific seizure and epilepsy models show a mixed spectrum of efficacy. It remains questionable whether fenfluramine would have been brought to clinical trials without further knowledge from clinical case series and data from etiology‐specific preclinical models.

The partially contrasting findings evident when comparing datasets from different laboratories could, as always, be related to differences between mouse and rat strains in terms of pharmacokinetics and pharmacodynamics as well as different pretreatment times. Unfortunately, efficacy data for fenfluramine and other ASMs are often not consistently reported along with exposure data, which would allow a better comparison across studies, strains and species.

### 2.2 Cannabidiol

Antiseizure effects of cannabidiol have been evaluated in various non‐specific acute seizure models and chronic epilepsy models. Recently, Del Pozo and Barker‐Haliski^115^ have published a comprehensive review summarizing and discussing the preclinical antiseizure profile across a variety of models. Thus, we refrain from repeating this information in detail and refer to the corresponding publication.^115^ However, we have updated the literature search and provide a brief overview of the preclinical efficacy profile of cannabidiol for the sake of completeness.

The majority of studies testing cannabidiol effects in the MES and PTZ tests and in strains with audiogenic seizure susceptibility reported antiseizure effects in mouse and rat models (Table 3).^23^, ^25^, ^33^, ^35^, ^37^, ^38^, ^45^, ^48^, ^49^, ^50^, ^53^, ^54^, ^58^, ^72^, ^73^, ^74^, ^93^ In this context, it needs to be considered that those studies that evaluated efficacy in relation to tolerability with a focus on motor dysfunction reported relatively low protective indices (Table 3).

A protection against PTZ‐induced seizures was also confirmed in a zebrafish paradigm.^60^, ^61^ In the 6‐Hz mouse model, protective effects of cannabidiol were observed even at high stimulation strengths, where various ASMs fail to exert effects at tolerated doses.^35^, ^65^

Moreover, testing of cannabidiol in different chemically‐induced *status epilepticus* (SE) models demonstrated a relevant efficacy.^84^, ^85^, ^87^, ^88^

Inconsistent findings have been reported from chronic chemical and electrical kindling paradigms with some but not all studies describing antiseizure effects and a delay in kindling progression (Table 3).^91^, ^92^, ^94^, ^98^ However, cannabidiol failed to show antiseizure efficacy in lamotrigine (LTG)–resistant kindled mice.^26^ Moreover, an antiseizure effect of cannabidiol was confirmed in different chemically induced post‐SE models (Table 3).^102^, ^104^

Although available preclinical findings do not provide consistent information about the antiseizure potential of cannabidiol, several studies provided valuable information about the impact of cannabidiol on seizure generation in the naive and the epileptic brain of laboratory rodents.

### 2.3 Stiripentol

Etiology‐independent antiseizure effects of stiripentol have been assessed in various seizure and epilepsy models.^20^, ^30^, ^31^, ^32^, ^44^, ^116^, ^117^ In the subcutaneous and the intravenous PTZ test and the MES test, protective antiseizure effects of stiripentol have been reproduced repeatedly in different studies of independent laboratories.^30^, ^31^, ^32^, ^44^, ^117^ A comparison of both enantiomers revealed that (+)‐stiripentol shows more potent protective effects against PTZ‐induced seizures.^116^

In terms of efficacy in a chronic epileptic state with assessment in brain slices from epileptic rats, stiripentol reduced the frequency of spontaneous epileptiform discharges but increased their duration and amplitude.^110^ An impact on spontaneous recurrent electroencephalographic seizures was not confirmed in an intrahippocampal kainate model in mice, in which stiripentol failed to reduce the frequency of paroxysmal discharges.^105^

In rat models of SE and refractory SE, stiripentol efficaciously terminated the prolonged seizure activity.^44^, ^80^, ^81^ In this context, it is emphasized that there is a general interest to assess prevention of SE development and termination of SE for screening of ASM candidates for DEEs, as several DEEs are associated with a high risk of repeated SE associated with frequent hospitalization and increased mortality risks. Unfortunately, preclinical data from SE models seem to be very limited for ASMs licensed for DEEs.

Of interest, these studies as well as an analysis in a PTZ test revealed that the efficacy in young rats exceeded that in young adult rats.^44^, ^80^ Moreover, acute seizure models (PTZ and MES) were not only used to confirm antiseizure effects of stiripentol, but also to explore the interaction of stiripentol with other ASMs based on isobolographic analysis.^30^, ^31^, ^52^ These studies provided information about possible sub‐additive (antagonistic), additive, and supra‐additive (synergistic) effects and their nature differentiating between pharmacodynamics and pharmacokinetic interactions. Some of the pharmacokinetic interactions have also been validated by clinical data and are also listed in the product information.^118^, ^119^, ^120^, ^121^ These, for example, comprise an increase in carbamazepine, phenobarbital, and clobazam concentrations and a reduction in the concentration of the metabolites of valproic acid.^118^, ^119^, ^120^, ^121^

In this context it is emphasized that pharmacokinetic interactions can largely differ between laboratory animals and patients due to species differences in metabolic pathways. Concerning pharmacodynamic synergism and antagonism, conclusions from clinical data are rather limited to observations and study data, suggesting beneficial combinations or unfavorable combinations. It is impossible to perform isobolographic analysis in the clinical setting in patients with DEEs. Thus, it is not possible to conclude about the informative value of respective preclinical data.

Early during preclinical development, stiripentol and its combination with carbamazepine were also tested in a rhesus monkey model with seizure induction by alumina‐gel injections.^122^ In this study, acute stiripentol exposure delayed the onset of seizures but did not eliminate seizure activity and chronic stiripentol exposure reduced electroencephalographic interictal spike rates. The authors concluded that the findings might indicate efficacy against generalized non‐motor (absence) seizures and that polytherapy approaches should be evaluated involving stiripentol.^122^, ^123^

Taken together, the majority of preclinical studies in rodent seizure and epilepsy models confirmed a promising antiseizure profile of stiripentol.

### 2.4 Everolimus

Everolimus is considered the first true disease‐targeting, precision medicine approach for any DEE. Despite its development and approval for therapeutic management of drug‐refractory epilepsy associated with tuberous sclerosis, various studies analyzed the efficacy of this drug in induced non‐specific seizure and epilepsy models. Assessment in the MEST test, which was conducted in naïve mice and in mice with spontaneous recurrent seizures (pilocarpine post‐SE model), revealed a general antiseizure effect of everolimus.^108^ In chronic intrahippocampal kainate models, everolimus did not exert robust antiseizure effects on recurrent electroencephalographic seizure events.^106^, ^107^ Moreover, everolimus failed to show preventive disease‐modifying or antiepileptogenic effects in an intraperitoneal kainate model in rats.^101^, ^103^

In summary, available data from testing of everolimus in non‐specific seizure and epilepsy models have a limited informative value not allowing robust conclusions about a possible broader spectrum potential.

### 2.5 Ganaxolone

Various studies have assessed ganaxolone's antiseizure effects in induced non‐specific seizure and epilepsy models. In the subcutaneous mouse or rat PTZ model, ganaxolone showed a robust efficacy, which was replicated in several studies.^21^, ^43^, ^46^, ^47^, ^56^ In one of these studies, chronic ganaxolone exposure data argued against self‐tolerance induction.^46^ Efficacy against acute seizures in mice was further confirmed in models with seizure induction by various other chemoconvulsants and in paradigms with electrical induction of acute seizure activity including the MES and the 6‐Hz test (32 mA).^21^, ^64^, ^67^

In kindling paradigms including PTZ, corneal, and hippocampal kindling approaches, different groups have assessed the efficacy of ganaxolone on seizures in fully kindled mice or on kindling progression.^21^, ^56^, ^66^, ^97^, ^99^, ^100^ In these studies, an antiseizure effect and a delay in kindling acquisition were reported.

In Wistar albino Glaxo from Rijswijk (WAG/Rij) rats, a model with spontaneous spike‐and‐wave discharges (SWDs) as the electroencephalographic hallmark of generalized non‐motor seizures, the effect of local ganaxolone administration largely differed depending on the target brain region with a suppression of SWDs following application into a subregion of the somatosensory cortex and an aggravation following application into thalamic regions.^71^

In mouse and rat models of treatment‐resistant SE, ganaxolone showed dose‐dependent effects on seizure activity.^82^, ^89^

Taken together, these preclinical data convincingly demonstrated a relevant antiseizure profile of ganaxolone.

### 2.6 Rufinamide

Antiseizure effects of rufinamide have been confirmed in various acute seizure models in mice and rats including the MES test, the 6‐Hz test (32 and 44 mA), the subcutaneous and intraperitoneal PTZ test, and further tests with administration of chemoconvulsants.^22^, ^24^, ^34^, ^57^ In contrast, no relevant efficacy was observed following administration of a combination with midazolam and ketamine in a soman‐induced SE model in rats.^83^ In a PTZ kindling paradigm, rufinamide exerted preventive effects delaying the progression of kindling acquisition.^96^ In contrast, the ASM failed to affect fully kindled seizures in LTG‐resistant kindled rats in a relevant manner.^90^

Thus, the preclinical efficacy profile of rufinamide does not provide a clear picture.

### 2.7 Non‐specific seizure and epilepsy models: informative value and limitations

**FIGURE 3:**
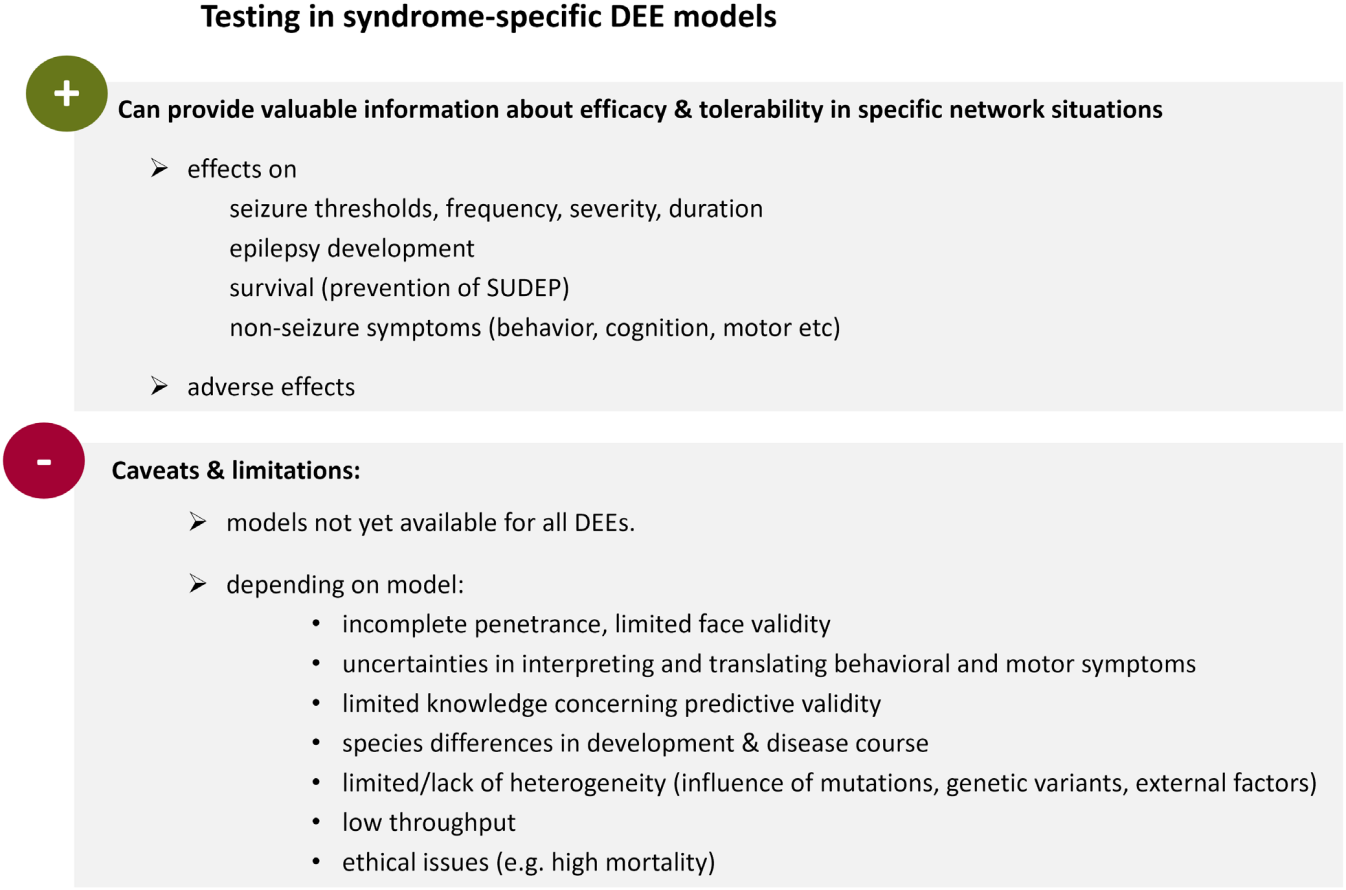
Opportunities, caveats, and limitations for testing drug candidates for developmental and epileptic encephalopathies (DEEs) in etiology‐ and syndrome‐specific models. SUDEP, sudden unexpected death in epilepsy.

As already stated, the application of screening programs with seizure and epilepsy models not specific for DEE etiologies and pathomechanisms, needs to carefully consider that these models do not recapitulate the disease‐specific network situation. Moreover, epileptogenic genetic variants not only influence the network situation, but can also cause complex secondary molecular and metabolic alterations,^124^, ^125^, ^126^ which might have implications for drug efficacy and tolerability. Another relevant limitation of these models is related to the fact that development of drugs for therapeutic management of DEEs should not only focus on anti‐seizure effects, but should carefully evaluate the impact of drugs on non‐seizure symptoms and on quality of life, as recently discussed for Dravet syndrome.^127^ The respective effects can be assessed only in specific DEE models that better reflect the characteristics of the entire disease spectrum, and thus allow determination of effects on cognition, behavior patterns including social interaction, and motor functions (e.g., Griffin et al.^128^) (Figure 3). Furthermore, an influence of drug candidates on the risk of SUDEP is of translational relevance. Conclusions require assessment in DEE models, which related to high seizure frequencies or to possible specific SUDEP mechanisms are often associated with a high seizure‐associated mortality rate. Finally, the timing of the start of therapy may be decisive when it comes to therapeutic success rates. Corresponding information can only be obtained from DEE models that recapitulate the age at onset and development course of the human disease as far as possible in a cross‐species translation. A relevant example has been the development of stiripentol for which a higher efficacy against hyperthermia‐induced seizure in a mouse model of Dravet syndrome has been reported in younger animals.^129^ This age dependency has been attributed to the expression peak of the alpha3 subunit of the GABA_A_ receptor during early brain development.^17^

Available preclinical datasets for licensed ASMs with proven clinical efficacy in selected DEEs confirm that testing in non‐specific seizure and epilepsy models may provide information about antiseizure effects of drug candidates, which are in development for DEEs. However, inconsistent findings that are evident for some ASMs already approved for selected DEEs underscore that these models may of course not help in selecting the best candidate for a particular DEE. As already discussed, these models cannot be used as the sole basis for decision‐making.

## 3 TESTING IN SYNDROME‐SPECIFIC DEVELOPMENTAL AND EPILEPTIC ENCEPHALOPATHY MODELS

As stated previously, syndrome‐specific DEE models better recapitulate the syndromic spectrum of symptoms and the syndrome‐related hyperexcitable neuronal network. Thus, testing in these models can provide valuable information about efficacy and tolerability in the syndrome‐specific network situations. In terms of efficacy, preclinical studies in DEE models can provide insight into the effects of ASMs and drug candidates on seizure thresholds, seizure frequency, severity and duration, epilepsy development, survival rates, and non‐seizure symptoms affecting cognition, behavior, and motor function. Moreover, testing scenarios can reflect different disease phases and associated seizure triggers. For genetic mouse models of Dravet syndrome, hyperthermia‐induced seizures became a frequently applied testing scenario,^130^ which has been recently integrated as a syndrome‐specific screening assay for Dravet syndrome in the National Institutes of Health (NIH)/National Institute of Neurological Disorders and Stroke (NINDS) Epilepsy Therapy Screening Project (ETSP).

### 3.1 Fenfluramine

The first preclinical confirmations for antiseizure effects of racemic fenfluramine in models of Dravet syndrome came from pharmacological analysis in an antisense knockdown zebrafish model and an *scn1a* mutant zebrafish model.^131^, ^132^ In the latter model, an impact on behavioral and electroencephalographic epileptiform activity was demonstrated, which was reduced by pretreatment with 5‐HT1D and 5‐HT2C receptor antagonists and a Sigma‐1 modulator.^16^ In the *Scn1a* A1783V knockin mouse model, fenfluramine failed to protect from hyperthermia‐induced seizures.^130^

Both, a zebrafish and a mouse model of Dravet syndrome provided insights into possible disease‐modifying effects of fenfluramine with an impact on dendritic arborization of GABAergic neurons and on cell hyperproliferation in *scn1a* mutant zebrafish and an impact on myelin damage, microglia activation, and apoptosis in an *Scn1a* ^+/−^ mouse model.^133^, ^134^ Finally, the model was also used to directly compare the efficacy of fenfluramine and norfenfluramine enantiomers confirming antiseizure effects of (+)‐ and (−)‐fenfluramine and (+)‐norfenfluramine.^135^

Taken together, available data from mouse and zebrafish models of Dravet syndrome provided convincing proof‐of‐principle for a relevant efficacy of fenfluramine in this DEE.

Concerning a possible future expansion of the indications, fenfluramine was also assessed in a mouse model of sodium voltage‐gated channel alpha subunit 8 (*Scn8a*)–related epilepsy with electrically triggered seizures.^63^ The study did not indicate a relevant efficacy in this genetic model.

### 3.2 Cannabidiol

As stated previously, Del Pozo and Barker‐Haliski^115^ already provide a comprehensive review presenting and discussing the preclinical efficacy profile of cannabidiol. Thus, we refer interested readers to the corresponding publication.^115^ However, we have updated the literature search and provide a brief overview of the preclinical efficacy profile of cannabidiol for the sake of completeness.

Testing in genetic zebrafish and mouse models of Dravet syndrome revealed a mixed efficacy pattern. Although cannabidiol affected spontaneous seizures in a mouse model, it failed to protect from hyperthermia‐induced seizures in a mouse model and spontaneous seizure events in the sodium channel, voltage‐gated, type I like, alpha b (*scn1Lab*) zebrafish model.^130^, ^135^, ^136^ Another zebrafish study demonstrated a reduction in locomotor activity in genetically‐modified animals and a reduction in PTZ‐induced changes in wild‐type fish.^62^ Moreover, a genetic mouse model was used to confirm the synergy between cannabidiol and clobazam.^137^

In summary, the efficacy data from mouse and zebrafish models of Dravet syndrome were not entirely consistent. Therefore, without knowledge of clinical experience, which was already available for cannabidiol from case reports and small case series, it seems as if further preclinical efforts would have been necessary to obtain more information on the efficacy of cannabidiol and to better understand the factors influencing efficacy before deciding to enter clinical studies.

A protective effect against PTZ‐induced seizures in mice with a cyclin‐dependent kinase like 5 (*CDKL5*) genetic variant^138^ provided preclinical evidence that cannabidiol might also exert beneficial effects in *CDKL5* deficiency disorder.

### 3.3 Stiripentol

In line with the licensing of stiripentol for adjunctive therapy of seizures in patients with Dravet syndrome, the efficacy of this ASM has been assessed in zebrafish and mouse models of Dravet syndrome.^129^, ^130^, ^139^, ^140^, ^141^, ^142^ In *scn1a* zebrafish mutants, stiripentol attenuated both, behavioral and electroencephalographic seizure correlates.^139^ In combination with clobazam, stiripentol caused a significant reduction in interictal spikes and an increase in the relative contribution of the beta frequency band in a Dravet mouse model (*Scn1a* ^A1783V/WT^).^140^ Aiming to develop an ASM screening platform for Dravet syndrome at the NINDS contract site for the ETSP, Pernici and colleagues (2021)^130^ have determined the response of hyperthermia‐induced seizures in the same Dravet syndrome mouse model (*Scn1a* ^A1783V/WT^). They demonstrated that hyperthermia‐induced seizures in this model are highly refractory to various ASMs. Concerning stiripentol, the study confirmed the efficacy of a combination with clobazam and valproic acid.^130^

Other studies failed to confirm relevant effects of stiripentol on hyperthermia‐induced seizure in genetic Dravet mouse models.^129^, ^141^ Of interest, one of these studies reported that a protective effect was only evident in younger mice (age 1 month), but not in 5‐month‐old Dravet mice.^129^

Taken together, preclinical data from mouse and zebrafish models of *Scn1a*‐related Dravet syndrome is in line with clinical efficacy data for stiripentol and its combinations with other ASMs in this DEE.

Considering that Dravet syndrome can also be associated with genetic variants of *GABRG2* encoding the γ2 subunit of the GABA_A_ receptor, Warner and colleagues explored spontaneous and PTZ‐induced seizures in Gabra^*+/Q390x*^ mice. In this genetic model, stiripentol increased the frequency of spontaneous and PTZ‐induced seizures and its combination with diazepam reduced the frequency of both seizure types.^142^

### 3.4 Everolimus

Applying the search string provided in the Supporting Information, we identified only a low number of preclinical studies evaluating everolimus in models of tuberous sclerosis complex or other DEEs.

A study in a mouse model with a cell‐specific conditional inactivation of the *Tsc1* gene in glial fibrillary acidic protein (GFAP)–expressing cells (*Tsc1* ^GFAP^‐Cre knockout) provided evidence for a good predictive validity of the genetic model as it captured efficacy of a novel selective mTORC1/2 inhibitor, a dual pan‐PI3K/mTORC1/2 inhibitor, and of rapamycin as a well characterized mTOR modulator.^106^ Moreover, testing of everolimus in brain slices from animals with a *Pik2ca* mutation and in *Tsc* ^+/−^ rats failed to confirm relevant antiseizure effects.^143^, ^144^

### 3.5 Ganaxolone

Applying the search string provided in the Supporting Information, we did not identify any preclinical studies evaluating ganaxolone in models of *CDKL5* deficiency disorder. However, testing in mouse models of Angelman syndrome and of fragile X syndrome with induction of audiogenic seizures and/or PTZ‐induced seizures demonstrated antiseizure effects.^145^, ^146^

### 3.6 Rufinamide

Applying the search string provided in the Supporting Information, we did not identify any preclinical studies evaluating rufinamide in models of Lennox–Gastaut syndrome. We identified only one study that evaluated the impact of rufinamide in a model with chronic administration of AY‐9944, which results in atypical absence seizures mimicking the respective seizure phenotype in Lennox–Gastaut syndrome.^57^ In a genetic Dravet mouse model, the assessment was limited to acute testing on hyperthermia‐induced seizures, which were not affected by rufinamide exposure.^130^

## 4 SYNDROME‐SPECIFIC DEVELOPMENTAL AND EPILEPTIC ENCEPHALOPATHY MODELS: INFORMATIVE VALUE, LIMITATIONS, AND CHALLENGES

Although syndrome‐specific DEE models often appear to be characterized by a good face and predictive validity, there can be limitations with an incomplete penetrance of seizure phenotypes in several models of DEEs (Figure 3).^147^ As discussed intensely by Bertocchi et al.,^147^ mice with a *Cdkl5* knockout or *Cdkl5* gene variant and mouse models of glutamate ionotropic receptor *N*‐methyl‐d‐aspartate (NMDA) (GRIN)‐related encephalopathies carrying mutations in genes encoding one of the NMDA receptor subunits represent examples of DEE models, which do not exhibit relevant seizure activity. Use of these and other models without a seizure phenotype for drug testing implies that chemical or noise triggers are necessary to induce seizure activity. As these triggers may affect the mechanisms of ictogenesis, these procedures can be theoretically associated with a potential bias and impact on predictive validity. Along this line, phenotypes are sometimes only evident in a homozygous situation as compared to heterozygosity in patients.^147^ This difference can also have implications for the network situation and its responsiveness to ASMs and drug candidates.

On the other hand, phenotypes can be rather severe and associated with high mortality rates resulting in ethical issues and high animal numbers needed.^147^ Moreover, the models are challenging to work with due to the intense care necessary to minimize mortality rates.

The time‐consuming nature of the work with genetic mouse models with spontaneous recurrent seizures is also related to the need for the continuous video‐electroencephalogram (EEG) monitoring necessary to assess the impact on seizure frequencies and seizure parameters. Although a spectrum of non‐seizure symptoms is observed in genetic DEE mouse models comprising possible alterations in mood, affective state, cognition, and alertness,^3^, ^128^, ^147^ there are remaining uncertainties when it comes to interpretation of behavioral patterns and translation to the clinical phenotypic spectrum. The same applies to the assessment of motor function, with readout parameters in mice as quadrupeds being less sensitive to syndrome‐related changes than bipeds. Finally, sleep analysis requires a thorough and very time‐consuming assessment based on continuous EEG/ electromyography (EMG) monitoring.

As evident from data summarized in Table 4, there is still limited knowledge concerning pharmacology and the predictive validity of the models and the testing scenarios. For example, several ASMs licensed for management of Dravet syndrome fail to exert relevant effects on hyperthermia‐induced seizures in mouse models of this DEE.^130^ It remains unclear whether hyperthermia‐induced seizures are characterized by a different pharmacology than recurrent seizures or whether the differences in efficacy are related to acute vs. chronic dosing regimens. Considering that one study in young mice with a genetic *Scn1a* deficiency demonstrated protection from hyperthermia‐induced seizures,^129^ further efforts seem necessary to determine factors (such as age, genetic variant, and so on) that might influence the response of hyperthermia‐induced seizure in different Dravet models to ASMs.

**TABLE 4:** Literature information: Efficacy of approved orphan drugs in etiology‐specific models of developmental and epileptic encephalopathies (DEEs).

DEE	Species: mutation	Antiseizure medications: Line 1*—*route of administration: direction of the effect; Line 2*—*comments/additional information					
		Fenfluramine	Stiripentol	Ganaxolone	Everolimus	Rufinamide	Cannabidiol
Dravet Syndrome	Zebrafish: *scn1Lab*	Medium: ↓^16^, ^131^, ^132^, ^135^	Medium: ↓^139^				Medium: ≈^135^
		Spontaneous	Spontaneous				‐
		Medium: ≈^132^	Medium: ≈^135^				Medium: ↓^62^
		Hyperthermia	Spontaneous				‐
	Mice: *Scn1a* ^A1783V/+^	i.p.: ≈^130^	i.p.: ≈^130^, ^140^			i.p.: ≈^130^	‐: ≈^130^
		Hyperthermia	Hyperthermia			Hyperthermia	Hyperthermia
	Mice: *Scn1a* ^E1099X/+^		i.p.: ≈^141^				
			Hyperthermia				
	Mice: Scn1a^+/−^ (exon 25)						i.p.: ↓^136^
							Hyperthermia/Seizure duration
	Mice: *Scn1a* ^+/−^ (exon 8)						i.p.: ↓^148^
							Θ‐γ coupling
	Mice: *Scn1a* ^+/−^ (exon 1)						i.p.: ↓/≈^137^, ^149^
							Hyperthermia
							p.o.: ≈^137^, ^149^
							Spontaneous
	Mice: *Scn1a* ^+/−^ (exon 26)						i.p.: ↓^150^
							Hyperthermia/Spontaneous
	Mice: *Scn1a* ^R1407X/+^		i.p.: ↓^129^				
			Hyperthermia/Young				
			i.p.: ≈^129^				
			Hyperthermia/Adult				
	Mice: *Gabrg2* ^Q390X/+^		i.p.: ↑^142^				
			Spontaneous				
			i.p.: ↑^142^				
			i.p. PTZ				
GABRA1 DEE	Zebrafish: *Gabra1* ^−/−^						Medium: ≈^61^
							Light‐induced
SCN8A DEE	Mice: *Scn8a* ^R1620L/+^	i.p.: ≈^63^					i.p.: ↓^151^
		6 Hz 16 mA					6 Hz 16/32 mA
							i.p.: ↓^151^
							s.c. PTZ
CDKL5 deficiency	Mice: *Cdkl5* ^R59X/+^						i.p.: ↓^138^
							i.p. PTZ
Pik3ca associated epilepsy	Mice: *Pik3ca* ^E545K/+^				Bath: ≈^143^		
					In vitro electrophysiology		
Tuberous Sclerosis Complex	Rats: *Tsc1* ^+/−^				i.p.: ≈^144^		
					Spontaneous		
Angelman syndrome	Mice: *Ube3a* ^tm1Alb^			s.c.: ↓^145^			
				Audiogenic			
				s.c.: ↓^145^			
				i.p. PTZ			
	Mice: Ube3a^m+/p−^						i.p.: ↓^152^
							Audiogenic
							i.p.: ≈^152^
							KDL
							i.p.: ↓^152^
							KDL + hyperthermia
Fragile X syndrome	Mice: *Frm1* ^−/−^			i.p.: ↓^146^			
				Audiogenic			
CLN disease	Mice: Cln^−/−^						p.o.: ≈^153^
							Spontaneous

*Note*: The direction of the effect is illustrated by: ↓ means a protective effect of the compound; ≈ means no evidence of a protective effects; ↑ means convulsive effects; ‐ means the lack of information or relevant comments provided. Please note the lack of information regarding median effective and toxic dose among these data.

Abbreviations: i.p., intraperitoneally; KDL, kindling; p.o., per os; PTZ, pentylenetetrazole; s.c., subcutaneously.

For DEEs other than Dravet, only very limited data from genetic rodent models are available (Table 4; Tables S2–S7), thus not providing a robust and solid basis for a retrospective evaluation of the predictive validity of these models.

In general, it is of particular interest to test for an association between time windows for therapy initiation and long‐term outcomes concerning potential disease‐modifying or preventive effects as well as differences in efficacy and tolerability during brain development and disease phases. The age‐dependent efficacy of stiripentol on hyperthermia‐induced seizures has already been discussed as a relevant practical example.^17^, ^129^ However, there are species‐related differences in the sequence of events during brain development that pose a challenge to direct translation of findings in mice to patients.

In this context, it is important to note that preclinical testing of drug candidates is often performed in young adult and adult animals, not recapitulating the initiation of therapy in infants and young children. This could lead to selection bias during drug development, implying that compounds with a favorable profile for neuropediatric therapy might be overlooked.

Finally, the time‐consuming nature of the preclinical studies in genetic models with spontaneous recurrent seizures often results in a limited heterogeneity or lack of heterogeneity in the testing scenarios. In this context it needs to be kept in mind that assessment in one genetic model with one selected genetic variant and a rather standardized genetic background and environment does not recapitulate the clinical variance related to gene variants, genetic modifiers, and numerous external factors. Although it would be highly informative in terms of robustness and generalizability of findings to test various doses in different models and laboratories, there are obvious limitations in terms of cost, time, and capacity. Thus testing scenarios in models of DEEs often at least partially neglect common recommendations to increase rigor and reproducibility and inform about the robustness and generalizability of preclinical data.^154^, ^155^

In comparison with mouse models, zebrafish models can allow a higher throughput; however, they have even more pronounced limitations when it comes to interspecies differences concerning the entire syndromic spectrum of symptoms or the developmental time course.^128^, ^156^ Nevertheless, concerning efficacy, available datasets support a relevant predictive validity of zebrafish models of Dravet syndrome confirming efficacy of ASMs in clinical use in patients with Dravet syndrome and lack of efficacy of ASMs that are contraindicated in this DEE.^128^, ^131^, ^139^, ^157^

Because various specific disease‐targeting approaches are in the drug development pipeline, it will be of particular interest to compare clinical outcomes with preclinical findings. First, clinical data from patients with *SCN1A*‐related Dravet syndrome seem to confirm a relevant efficacy of the antisense oligonucleotide zorevunersen (former STK001),^158^, ^159^ which mediates a targeted augmentation of nuclear gene output with increased production of the productive *SCN1A* and functional messenger RNA (mRNA) by modulation of a splicing event. These data, which resulted in the U.S. Food and Drug Administration (FDA) classification as a breakthrough product, are in line with earlier reports from a genetic mouse model of Dravet syndrome.^7^ An adeno‐associated viral vector‐based approach (ETX101) with cell‐selective expression of a transcription factor upregulating *SCN1A* expression has entered clinical testing in patients with Dravet syndrome in 2024. First reports at the company's website are limited to statements on tolerability (NCT06112275, NCT06283212, NCT05419492, https://encoded.com/press‐releases/encoded‐therapeutics‐reports‐clinical‐progress‐of‐etx101‐gene‐therapy‐for‐dravet‐syndrome‐recaps‐2024‐corporate‐achievements‐and‐provides‐2025‐outlook/). Once efficacy data become available, it will be interesting to compare them with preclinical data from a genetic mouse model of Dravet syndrome.^8^ Encouraged by promising preclinical data from a *SCN2A*‐GOF mouse model, the gapmer antisense oligonucleotide elsunersen (former PRAX‐222) has entered clinical testing in patients with the corresponding DEE. Recent clinical data have been published providing first evidence for successful translation to clinical use.^160^, ^161^ As conclusions about the efficacy of these and many other approaches can only be drawn once more comprehensive clinical datasets are available, it is too early to conclude about the predictive validity of DEE models for efficacy and tolerability testing of disease‐targeting approaches.

In this context, it is important to keep in mind that species differences might imply the need for a species‐specific design of candidates when it comes to *ATMPs*, including *GTMPs*, thereby increasing the effort and eventually limiting the translational value of the findings.

## 5 TESTING IN TRANSFECTED/TRANSDUCED CELLS, PATIENT‐DERIVED INDUCIBLE‐PLURIPOTENT STEM CELLS, OR BRAIN ORGANOIDS

It is emphasized that the assessment of the impact of specific genetic variants and of genetic modifiers can be of particular relevance as a basis for inclusion and exclusion criteria for clinical studies analyzing efficacy in patients with DEEs and for identifying the patient sub‐population that is likely to respond to a novel therapeutic approach. In addition to differentiation between loss‐of‐function and gain‐of‐function variants, specific genetic variants may alter affinity and or intrinsic activity of small molecules that selectively target a mutant protein. Novel therapeutic approaches targeting expression rates in patients with haploinsufficiencies need to take into account that some mutant proteins can exert dominant‐negative effects and, for instance, interfere with the processing, trafficking, or the function of the wild‐type protein.^162^ In these cases, non‐selectively upregulating expression of the mutant and wild‐type protein might fail to exert the expected therapeutic effects.

**FIGURE 4:**
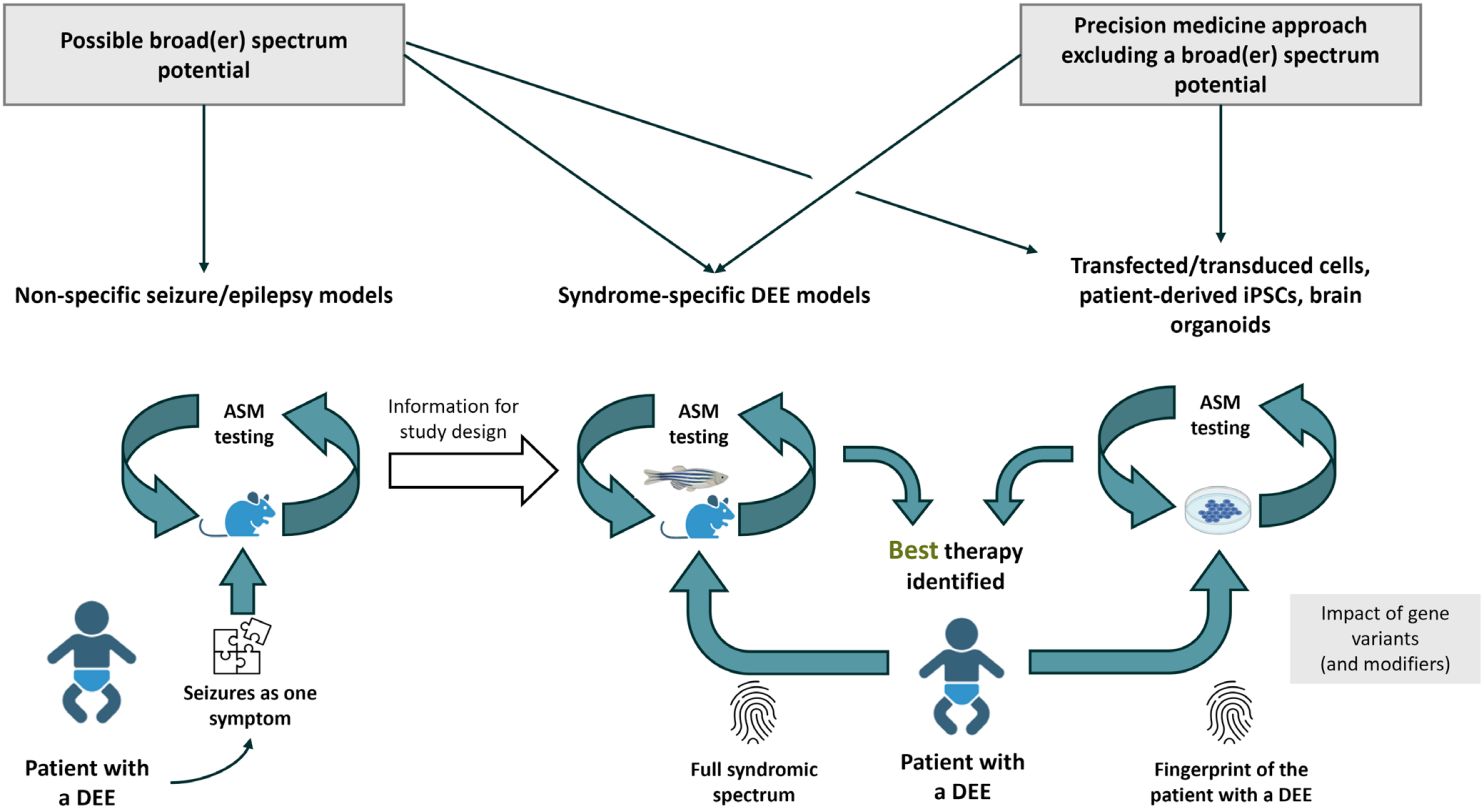
Preclinical testing strategies for drug candidates for developmental and epileptic encephalopathies (DEEs). Possible workflows with sequential and combined use of standard animal models, etiology‐/syndrome‐specific animal models, and cell‐ or organoid‐based assays in the discovery of effective antiseizure medications (ASMs). iPSCs, inducible‐pluripotent stem cells. Created in BioRender. Potschka, H. (2025) https://BioRender.com/a73f778. Figure created with BioRender.com.

Thus, testing in transfected cells or, even more importantly, in patient‐derived inducible‐pluripotent stem cells (iPSCs) or brain organoids is of particular relevance to narrow down the patient target group and optimize translational success rates (Figure 4). Although we have focused this review on the assessment of pharmacological therapeutic approaches for DEEs in animal models, we would like to emphasize the utmost importance of integrating appropriate human cell‐ and/or organoid‐based approaches as core elements of Investigational New Drug (IND)–enabling preclinical programs and to inform about inclusion and exclusion criteria for clinical trials.

For information about techniques and methods and a discussion of their informative value and limitations, we refer readers to excellent and comprehensive reviews and book chapters (e.g., by Varela et al.^163^; Hirose et al.^164^; Metcalf et al.^165^; Zayat et al.^166^; and Miguel Sanz et al.^167^).

### 5.1 Future perspectives and conclusions

Preclinical drug testing strategies for therapeutic approaches in the development for DEEs need to be carefully tailored considering the pharmacodynamic and pharmacokinetic characteristics of the drug candidate or other approach.

Well‐characterized etiology‐specific models of DEEs can provide a basis for drug candidate selection and decision‐making during preclinical development.

Thus, there is a particular need to expand our current state‐of‐knowledge concerning the predictive validity of available models of DEEs, considering the different testing scenarios in these models and aiming to identify experimental factors that influence responsiveness. The risk for false positives and negatives should be carefully evaluated in different DEE models. Depending on the stage of development—from lead discovery to lead optimization to more in‐depth preclinical testing—it is of course important to weigh intermediate/high‐throughput approaches (e.g., hyperthermia or chemical seizure induction in genetic DEE model) vs approaches providing more detailed and comprehensive information (e.g., chronic testing with 24/7 seizure monitoring plus analysis of behavior, cognition, and motor function).

The integration of corresponding models and study designs in preclinical screening and testing platforms requires a comprehensive knowledge about the face and predictive validity as far as this is possible based on available clinically validated therapies and comparison of preclinical and clinical effects on the full syndromic spectrum of symptoms of DEEs. The claim that a particular model and testing approach responds poorly to approved ASMs and is therefore well suited as a screening tool to select superior ASMs should—as far as this is possible—prompts intensive efforts to compare the mechanisms of drug resistance in the animal model with those in patients.

At the present time, we must admit that it remains unclear whether preclinical efficacy and tolerability testing in specific DEE models is of relevant informative value and therefore worth an investment in addition to efficacy testing in transfected/transduced cells, patient‐derived cells, and/or brain organoids.

Cumulating preclinical and clinical evidence for efficacy of different GTMPs and other disease‐targeting approaches for Dravet syndrome and some other specific DEEs, is expected to trigger a wave of corresponding developments for various DEEs. In this context, it is evident that we are not yet well prepared for preclinical in vivo testing, and therefore need to intensify our efforts to develop further genetic models of different DEEs, which show a good penetrance of phenotypes, and to characterize these models comprehensively. Despite the evident gaps‐in‐knowledge, evidence exists that some genetic models might allow prediction of efficacy/tolerability ratios not only focused on antiseizure effects but also the full syndromic spectrum of symptoms contributing to the burden of DEEs.

## AUTHOR CONTRIBUTIONS

Heidrun Potschka: Conceptualization (lead) methodology (supporting), visualization (equal), writing——original draft preparation (lead), and writing—review and editing (lead). Daniel Perez‐Perez: Methodology (lead), visualization (equal), writing—original draft preparation (supporting), and writing—review and editing (supporting).

## CONFLICT OF INTEREST STATEMENT

H.P. received fees and funding for consulting, presentations, and/or research collaborations from Lario/Exeed Epidarex, Jazz Pharmaceuticals, Angelini Pharma, Bayer, Eisai, Elanco, Galapagos, Jazz Pharmaceuticals, MSD, Zogenix, Bial, and Roche. The remaining authors have no conflicts of interest. We confirm that we have read the Journal's position on issues involved in ethical publication and affirm that this report is consistent with those guidelines.

## Supporting information


Data S1.

## Data Availability

Data sharing is not applicable to this article as no new data were created or analyzed in this study.
