# Supplementary material for: Drug discovery and preclinical testing of drug candidates for developmental and epileptic encephalopathies
Source: Epilepsia. 2025 Aug 6;66(12):4597–623. doi: 10.1111/epi.18581 (PMC12779334; doi:10.1111/epi.18581)
Supplement: Supplementary file 1 — Data S1. [file EPI-66-4597-s001.docx]

**Drug discovery and preclinical testing of drug candidates for developmental and epileptic encephalopathies**

Heidrun Potschka and Daniel Pérez-Pérez

**Supplementary information**

*Supplementary materials and methods*

*Supplementary references*

*Supplementary tables*

**Supplementary materials and methods**

This review is based on a systematized approach applied for identification of studies with preclinical pharmacological evaluation of antiseizure medications (ASMs) in *in-vivo* models of seizures or epilepsy. Concerning non-specific seizure and epilepsy models the search was focused on models currently integrated in the Epilepsy Therapy Screening Program of the National Institutes of Health (https://panache.ninds.nih.gov/Home/CurrentModels). However, further less common models were also considered.

The search focused on ASMs licensed for developmental and epileptic encephalopathies (DEEs). We excluded reviews, editorial comments, corrections, and human or veterinary clinical trials. The search was limited to the PubMed® data base and all information published before September 2024. Publications and references from the personal library of the authors and not necessarily listed in PubMed® were also included. In addition, reference lists of the included papers were screened to identify further relevant publications.

*Search strategy.*

We combined the relevant search terms for every compound and the relevant search terms for the different models of epilepsy, seizures, or DEEs. We developed search strings for the compounds of interest in this review: “fenfluramine”, “stiripentol”, “ganaxolone”, “cannabidiol”, “everolimus”, and “rufinamide” (Table S1). To identify models of acute seizures, we combined the following concepts: “acute seizure models”, “maximal electroshock seizure”, “maximal electroshock seizure threshold”, “6-Hz stimulation”, “subcutaneous pentylenetetrazole”, “intravenous pentylenetetrazole”, “status epilepticus”, and “benzodiazepine-resistant status epilepticus” (Table S1). For the chronic models of seizures or epilepsy, we combined “chronic seizure models”, “corneal kindling”, “intrahippocampal kainate model”, “intraamygdala kainate model”, “lamotrigine-resistant kindling model”, “drug resistant epilepsy models”, and “post-status epilepticus epilepsy models” (Table S1). In addition, we included “zebrafish” in combination with “seizure models” to identify relevant literature in this species (Table S1). To identify DEE-related models we combined “DEEs” and “animal models”, this search was then combined with the listed compounds (Table S1). If the compound is currently on use for one DEE, we looked for the relevant preclinical information on that DEE for that compound. Boolean operators and wild cards were used to increase the reach of the search.

Each search consisted of joining the compound strings (IDs 1-6) with the concepts related to the seizure models, either the acute seizure models (IDs 7-12), chronic seizure models (IDs 13-19), zebrafish models (ID 20 + ID26), or DEE models (IDs 21-25 + ID26). The two concepts were joined by using the connector “AND”. For the zebrafish and DEE models, the search was supplemented with the auxiliary “animal models” concept (ID 26) joined by the “AND” operator.

*Non-specific seizure and epilepsy models*

For information on predictive validity readers are referred to Table 1. In the following we provide a short description about the technical procedure and readout parameters for the most common non-specific models.

**Maximal Electroshock Seizure (MES):** This model is based on suprathreshold electrical stimulation (e.g. via corneal or ear electrodes) inducing generalized tonic-clonic seizures. Common data analysis approaches focus on % animals protected from seizure induction. ^1^

**Maximum electroshock seizure threshold test (MEST):** This model is a variant of the MES model, which is based on a staircase up-and-down stimulation procedure during which the stimulation current in individual animals is adjusted depending on seizure development in the previous animal of a group. This approach allows the determination of a seizure threshold for a group of animals. ^1^

**6-Hz test:** This model has been discarded in earlier days because of the lack of efficacy of ASMs such as phenytoin. In the search for screening models that enable better selection of drug candidates with superior efficacy in difficult-to-treat or drug-refractory epilepsies, this model has been reactivated and intensively characterized. Low-frequency electrical corneal stimulation of mice or rats triggers psychomotor seizures typically characterized by a minimal clonic phase followed by stereotyped behavioral patterns. ^2, 3^ At high stimulation intensities (44 mA) many ASMs do not exert relevant antiseizure effects in this model.

**Pentylenetetrazol (PTZ)-test:** This model is based on administration of the chemoconvulsant PTZ, which acts as a GABA_A_ receptor antagonist. Administration of PTZ triggers generalized tonic-clonic seizures in mice and rats. ^4^ While subcutaneous administrations allows assessment of latency to seizure onset, severity and duration of seizure activity, intravenous administration of PTZ by infusion provides a basis to assess seizure thresholds based on the PTZ dose. Interpretation of findings should consider pharmacokinetic interactions of test compounds with PTZ and a possible bias related to the specific mechanism of seizure induction by the chemoconvulsant. ^1^

**Kindling model.** The model is based on repeated electrical (e.g. corneal or via depth hippocampal or amygdalar depth electrodes) or chemical (e.g. PTZ) induction of seizures evolving in terms of severity and duration with ongoing stimulations. Kindled mice or rats develop a hyperexcitable network with lowered seizure thresholds as a consequence of the kindling procedure. Drug testing in so called fully kindled animals, which reproducibly exhibit generalized clonic seizures in response to stimulation, allows to determine seizure thresholds, seizure severity, and duration. Moreover, administration of test compounds during the kindling procedure allows to assess a possible impact on the generation of a hyperexcitable kindled network. ^5^

**Post-SE epilepsy models**. These mouse or rat models are based on chemical (e.g. pilocarpine, kainic acid) or electrical (e.g. via depth electrodes in the amygdala or hippocampus) induction of prolonged seizure activity. The associated brain insult triggers the development of epilepsy with spontaneous recurrent seizures. ^5^ The latency period differs depending on the model. Among these models the **intrahippocampal kainic acid model of mesial temporal lobe epilepsy** is frequently used as it is characterized by highly frequent electrographic seizure events in the chronic phase, which show a poor response to many ASMs. ^6^

Supplementary references.

1. Löscher W, Fassbender CP, Nolting B. The role of technical, biological and pharmacological factors in the laboratory evaluation of anticonvulsant drugs. II. Maximal electroshock seizure models Epilepsy Res. 1991 Mar;8:79-94.

2. Barton ME, Klein BD, Wolf HH, White HS. Pharmacological characterization of the 6 Hz psychomotor seizure model of partial epilepsy Epilepsy Res. 2001 Dec;47:217-227.

3. Metcalf CS, West PJ, Thomson KE, Edwards SF, Smith MD, White HS, et al. Development and pharmacologic characterization of the rat 6 Hz model of partial seizures Epilepsia. 2017 Jun;58:1073-1084.

4. White HS, Johnson M, Wolf HH, Kupferberg HJ. The early identification of anticonvulsant activity: role of the maximal electroshock and subcutaneous pentylenetetrazol seizure models Ital J Neurol Sci. 1995 Feb-Mar;16:73-77.

5. Löscher W. Animal models of epilepsy for the development of antiepileptogenic and disease-modifying drugs. A comparison of the pharmacology of kindling and post-status epilepticus models of temporal lobe epilepsy Epilepsy Res. 2002 Jun;50:105-123.

6. Duveau V, Pouyatos B, Bressand K, Bouyssieres C, Chabrol T, Roche Y, et al. Differential Effects of Antiepileptic Drugs on Focal Seizures in the Intrahippocampal Kainate Mouse Model of Mesial Temporal Lobe Epilepsy CNS Neurosci Ther. 2016 Jun;22:497-506.

7. Lazarova M, Samanin R. Serotonin mediation of the protective effect of clonidine against pentylenetetrazol-induced seizures in rats Life Sci. 1983 May 16;32:2343-2348.

8. Löscher W. Influence of pharmacological manipulation of inhibitory and excitatory neurotransmitter systems on seizure behavior in the Mongolian gerbil J Pharmacol Exp Ther. 1985 Apr;233:204-213.

9. Gentsch K, Heinemann U, Schmitz B, Behr J. Fenfluramine blocks low-Mg2+-induced epileptiform activity in rat entorhinal cortex Epilepsia. 2000 Aug;41:925-928.

10. Silenieks LB, Carroll NK, Van Niekerk A, Van Niekerk E, Taylor C, Upton N, et al. Evaluation of Selective 5-HT(2C) Agonists in Acute Seizure Models ACS Chem Neurosci. 2019 Jul 17;10:3284-3295.

11. Martin P, White HS, Barker-Haliski M. Evaluation of the Acute Anticonvulsant Efficacy of Fenfluramine in Mouse Models of Acute and Chronic Seizures. American Epilepsy Society Annual Meeting; 8/12/2019; Maryland, United States of America2019.

12. Wong JC, Escayg A. Carvedilol increases seizure resistance in a mouse model of SCN8A-derived epilepsy Front Pharmacol. 2024;15:1397225.

13. Tupal S, Faingold CL. Fenfluramine, a serotonin-releasing drug, prevents seizure-induced respiratory arrest and is anticonvulsant in the DBA/1 mouse model of SUDEP Epilepsia. 2019 Mar;60:485-494.

14. Tupal S, Faingold CL. Serotonin 5-HT(4) receptors play a critical role in the action of fenfluramine to block seizure-induced sudden death in a mouse model of SUDEP Epilepsy Res. 2021 Nov;177:106777.

15. Okanari K, Teranishi H, Umeda R, Shikano K, Inoue M, Hanada T, et al. Behavioral and neurotransmitter changes on antiepileptic drugs treatment in the zebrafish pentylenetetrazol-induced seizure model Behav Brain Res. 2024 Apr 27;464:114920.

16. Erenburg N, Hamed R, Shaul C, Perucca E, Bialer M. Comparative activity of the enantiomers of fenfluramine and norfenfluramine in rodent seizure models, and relationship with their concentrations in plasma and brain Epilepsia. 2023 Jun;64:1673-1683.

17. Erenburg N, Perucca E, Bechard J, Dube C, Weishaupt N, Sherrington R, et al. Stereoselective Analysis of the Antiseizure Activity of Fenfluramine and Norfenfluramine in Mice: Is l-Norfenfluramine a Better Follow-Up Compound to Racemic-Fenfluramine? Int J Mol Sci. 2024 Feb 21;25.

18. Pérez‐Pérez D, Monío‐Baca C, Von Rüden EL, Buchecker V, Wagner A, Schönhoff K, et al. Preclinical efficacy profiles of the sigma‐1 modulator E1R and of fenfluramine in two chronic mouse epilepsy models Epilepsia. 2024.

19. Rodriguez-Munoz M, Sanchez-Blazquez P, Garzon J. Fenfluramine diminishes NMDA receptor-mediated seizures via its mixed activity at serotonin 5HT2A and type 1 sigma receptors Oncotarget. 2018 May 4;9:23373-23389.

20. Sourbron J, Schneider H, Kecskes A, Liu Y, Buening EM, Lagae L, et al. Serotonergic Modulation as Effective Treatment for Dravet Syndrome in a Zebrafish Mutant Model ACS Chem Neurosci. 2016 May 18;7:588-598.

21. Zhang Y, Kecskes A, Copmans D, Langlois M, Crawford AD, Ceulemans B, et al. Pharmacological characterization of an antisense knockdown zebrafish model of Dravet syndrome: inhibition of epileptic seizures by the serotonin agonist fenfluramine PLoS One. 2015;10:e0125898.

22. Sourbron J, Smolders I, de Witte P, Lagae L. Pharmacological Analysis of the Anti-epileptic Mechanisms of Fenfluramine in scn1a Mutant Zebrafish Front Pharmacol. 2017;8:191.

23. Li J, Nelis M, Sourbron J, Copmans D, Lagae L, Cabooter D, et al. Efficacy of Fenfluramine and Norfenfluramine Enantiomers and Various Antiepileptic Drugs in a Zebrafish Model of Dravet Syndrome Neurochem Res. 2021 Sep;46:2249-2261.

24. Pernici CD, Mensah JA, Dahle EJ, Johnson KJ, Handy L, Buxton L, et al. Development of an antiseizure drug screening platform for Dravet syndrome at the NINDS contract site for the Epilepsy Therapy Screening Program Epilepsia. 2021;62:1665-1676.

25. Auvin S, Lecointe C, Dupuis N, Desnous B, Lebon S, Gressens P, et al. Stiripentol exhibits higher anticonvulsant properties in the immature than in the mature rat brain Epilepsia. 2013;54:2082-2090.

26. Cuan Y, He X, Zhao Y, Yang J, Bai Y, Sun Y, et al. Anticonvulsant Activity of Halogen-Substituted Cinnamic Acid Derivatives and Their Effects on Glycosylation of PTZ-Induced Chronic Epilepsy in Mice Molecules. 2017;23:76.

27. Luszczki J, Trojnar MK, Ratnaraj N, Patsalos PN, Czuczwar SJ. Interactions of stiripentol with clobazam and valproate in the mouse maximal electroshock-induced seizure model Epilepsy Res. 2010 Aug;90:188-198.

28. Luszczki J, Ratnaraj N, Patsalos PN, Czuczwar SJ. Characterization of the anticonvulsant, behavioral and pharmacokinetic interaction profiles of stiripentol in combination with clonazepam, ethosuximide, phenobarbital, and valproate using isobolographic analysis Epilepsia. 2006 Nov;47:1841-1854.

29. Luszczki J, Czuczwar SJ. Biphasic characteristic of interactions between stiripentol and carbamazepine in the mouse maximal electroshock-induced seizure model: a three-dimensional isobolographic analysis Naunyn Schmiedebergs Arch Pharmacol. 2006 Oct;374:51-64.

30. Shen DD, Levy RH, Moor MJ, Savitch JL. Efficacy of stiripentol in the intravenous pentylenetetrazol infusion seizure model in the rat Epilepsy Res. 1990 Sep-Oct;7:40-48.

31. Sada N, Lee S, Katsu T, Otsuki T, Inoue T. Epilepsy treatment. Targeting LDH enzymes with a stiripentol analog to treat epilepsy Science. 2015 Mar 20;347:1362-1367.

32. Grosenbaugh DK, Mott DD. Stiripentol in refractory status epilepticus Epilepsia. 2013 Sep;54 Suppl 6:103-105.

33. Grosenbaugh DK, Mott DD. Stiripentol is anticonvulsant by potentiating GABAergic transmission in a model of benzodiazepine-refractory status epilepticus Neuropharmacology. 2013 Apr;67:136-143.

34. Gasior M, Ungard JT, Beekman M, Carter RB, Witkin JM. Acute and chronic effects of the synthetic neuroactive steroid, ganaxolone, against the convulsive and lethal effects of pentylenetetrazol in seizure-kindled mice: comparison with diazepam and valproate Neuropharmacology. 2000 Apr 27;39:1184-1196.

35. West PJ, Saunders GW, Billingsley P, Smith MD, White HS, Metcalf CS, et al. Recurrent epileptiform discharges in the medial entorhinal cortex of kainate-treated rats are differentially sensitive to antiseizure drugs Epilepsia. 2018 Nov;59:2035-2048.

36. Girard P, Bacq A, Cloarec P, Lesueur C, Verleye M, Castagne V. Stiripentol efficacy against status epilepticus and associated mortality in mice Heliyon. 2024 Aug 15;10:e34854.

37. Riban V, Heulard I, Reversat L, Si Hocine H, Verleye M. Stiripentol inhibits spike-and-wave discharges in animal models of absence seizures: A new mechanism of action involving T-type calcium channels Epilepsia. 2022 May;63:1200-1210.

38. Poisson M, Huguet F, Savattier A, Bakri-Logeais F, Narcisse G. A new type of anticonvulsant, stiripentol. Pharmacological profile and neurochemical study Arzneimittelforschung. 1984;34:199-204.

39. Baraban SC, Dinday MT, Hortopan GA. Drug screening in Scn1a zebrafish mutant identifies clemizole as a potential Dravet syndrome treatment Nat Commun. 2013;4:2410.

40. Quinn S, Brusel M, Ovadia M, Rubinstein M. Acute effect of antiseizure drugs on background oscillations in Scn1a (A1783V) Dravet syndrome mouse model Front Pharmacol. 2023;14:1118216.

41. Ho SY, Lin L, Chen IC, Tsai CW, Chang FC, Liou HH. Perampanel Reduces Hyperthermia-Induced Seizures in Dravet Syndrome Mouse Model Front Pharmacol. 2021;12:682767.

42. Cao D, Ohtani H, Ogiwara I, Ohtani S, Takahashi Y, Yamakawa K, et al. Efficacy of stiripentol in hyperthermia‐induced seizures in a mouse model of Dravet syndrome Epilepsia. 2012;53:1140-1145.

43. Warner TA, Smith NK, Kang JQ. The therapeutic effect of stiripentol in Gabrg2(+/Q390X) mice associated with epileptic encephalopathy Epilepsy Res. 2019 Aug;154:8-12.

44. Li J, Sha L, Xu Q. Long-term outcomes of classic and novel anti-seizure medication in a kainate-induced model of chronic epilepsy Epilepsy Res. 2023 Mar;191:107095.

45. Theilmann W, Gericke B, Schidlitzki A, Muneeb Anjum SM, Borsdorf S, Harries T, et al. Novel brain permeant mTORC1/2 inhibitors are as efficacious as rapamycin or everolimus in mouse models of acquired partial epilepsy and tuberous sclerosis complex Neuropharmacology. 2020 Dec 1;180:108297.

46. Brandt C, Hillmann P, Noack A, Romermann K, Ohler LA, Rageot D, et al. The novel, catalytic mTORC1/2 inhibitor PQR620 and the PI3K/mTORC1/2 inhibitor PQR530 effectively cross the blood-brain barrier and increase seizure threshold in a mouse model of chronic epilepsy Neuropharmacology. 2018 Sep 15;140:107-120.

47. Barker-Haliski M, Knox K, Zierath D, Koneval Z, Metcalf C, Wilcox KS, et al. Development of an antiepileptogenesis drug screening platform: Effects of everolimus and phenobarbital Epilepsia. 2021 Jul;62:1677-1688.

48. Petrasek T, Vojtechova I, Klovrza O, Tuckova K, Vejmola C, Rak J, et al. mTOR inhibitor improves autistic-like behaviors related to Tsc2 haploinsufficiency but not following developmental status epilepticus J Neurodev Disord. 2021 Apr 17;13:14.

49. Thomson KE, Metcalf CS, Newell TG, Huff J, Edwards SF, West PJ, et al. Evaluation of subchronic administration of antiseizure drugs in spontaneously seizing rats Epilepsia. 2020 Jun;61:1301-1311.

50. Roy A, Han VZ, Bard AM, Wehle DT, Smith SEP, Ramirez JM, et al. Non-synaptic Cell-Autonomous Mechanisms Underlie Neuronal Hyperactivity in a Genetic Model of PIK3CA-Driven Intractable Epilepsy Front Mol Neurosci. 2021;14:772847.

51. Chuang SH, Reddy DS. Isobolographic Analysis of Antiseizure Activity of the GABA Type A Receptor-Modulating Synthetic Neurosteroids Brexanolone and Ganaxolone with Tiagabine and Midazolam J Pharmacol Exp Ther. 2020 Mar;372:285-298.

52. Zolkowska D, Wu CY, Rogawski MA. Intramuscular allopregnanolone and ganaxolone in a mouse model of treatment-resistant status epilepticus Epilepsia. 2018 Oct;59 Suppl 2:220-227.

53. Yum MS, Lee M, Ko TS, Velisek L. A potential effect of ganaxolone in an animal model of infantile spasms Epilepsy Res. 2014 Nov;108:1492-1500.

54. Reddy DS, Rogawski MA. Ganaxolone suppression of behavioral and electrographic seizures in the mouse amygdala kindling model Epilepsy Res. 2010 May;89:254-260.

55. Mares P, Stehlikova M. Anticonvulsant doses of ganaxolone do not compromise motor performance in immature rats Neurosci Lett. 2010 Jan 29;469:396-399.

56. Citraro R, Russo E, Di Paola ED, Ibbadu GF, Gratteri S, Marra R, et al. Effects of some neurosteroids injected into some brain areas of WAG/Rij rats, an animal model of generalized absence epilepsy Neuropharmacology. 2006 Jun;50:1059-1071.

57. Kaminski RM, Livingood MR, Rogawski MA. Allopregnanolone analogs that positively modulate GABA receptors protect against partial seizures induced by 6-Hz electrical stimulation in mice Epilepsia. 2004 Jul;45:864-867.

58. Reddy DS, Rogawski MA. Enhanced anticonvulsant activity of ganaxolone after neurosteroid withdrawal in a rat model of catamenial epilepsy J Pharmacol Exp Ther. 2000 Sep;294:909-915.

59. Liptakova S, Velisek L, Veliskova J, Moshe SL. Effect of ganaxolone on flurothyl seizures in developing rats Epilepsia. 2000 Jul;41:788-793.

60. Miller SL, Bennet L, Sutherland AE, Pham Y, McDonald C, Castillo-Melendez M, et al. Ganaxolone versus Phenobarbital for Neonatal Seizure Management Ann Neurol. 2022 Dec;92:1066-1079.

61. Reddy DS, Carver CM, Clossen B, Wu X. Extrasynaptic gamma-aminobutyric acid type A receptor-mediated sex differences in the antiseizure activity of neurosteroids in status epilepticus and complex partial seizures Epilepsia. 2019 Apr;60:730-743.

62. Reddy DS, Yoshimura RF, Ramanathan G, Carver C, Johnstone TB, Hogenkamp DJ, et al. Role of beta(2/3)-specific GABA-A receptor isoforms in the development of hippocampus kindling epileptogenesis Epilepsy Behav. 2018 May;82:57-63.

63. Beekman M, Ungard JT, Gasior M, Carter RB, Dijkstra D, Goldberg SR, et al. Reversal of behavioral effects of pentylenetetrazol by the neuroactive steroid ganaxolone J Pharmacol Exp Ther. 1998 Mar;284:868-877.

64. Carter RB, Wood PL, Wieland S, Hawkinson JE, Belelli D, Lambert JJ, et al. Characterization of the anticonvulsant properties of ganaxolone (CCD 1042; 3alpha-hydroxy-3beta-methyl-5alpha-pregnan-20-one), a selective, high-affinity, steroid modulator of the gamma-aminobutyric acid(A) receptor J Pharmacol Exp Ther. 1997 Mar;280:1284-1295.

65. Mares P, Kubova H, Kasal A. Anticonvulsant action of a new analogue of allopregnanolone in immature rats Physiol Res. 2010;59:305-308.

66. Reddy DS, Rogawski MA. Chronic treatment with the neuroactive steroid ganaxolone in the rat induces anticonvulsant tolerance to diazepam but not to itself J Pharmacol Exp Ther. 2000 Dec;295:1241-1248.

67. Saporito MS, Gruner JA, DiCamillo A, Hinchliffe R, Barker-Haliski M, White HS. Intravenously Administered Ganaxolone Blocks Diazepam-Resistant Lithium-Pilocarpine-Induced Status Epilepticus in Rats: Comparison with Allopregnanolone J Pharmacol Exp Ther. 2019 Mar;368:326-337.

68. Heulens I, D'Hulst C, Van Dam D, De Deyn PP, Kooy RF. Pharmacological treatment of fragile X syndrome with GABAergic drugs in a knockout mouse model Behav Brain Res. 2012 Apr 1;229:244-249.

69. Ciarlone SL, Wang X, Rogawski MA, Weeber EJ. Effects of the synthetic neurosteroid ganaxolone on seizure activity and behavioral deficits in an Angelman syndrome mouse model Neuropharmacology. 2017 Apr;116:142-150.

70. Sabir A, Tehreem S, Farooq M, Ashraf W, Javaid S, Ahmad T, et al. Evaluation of ivermectin and vitamin E based combination with antiseizure rufinamide drug for mitigation of pentylenetetrazole-induced kindling, behavioral challenges and histopathological aberrations J Physiol Pharmacol. 2024 Jun;75.

71. Lumley LA, Nguyen DA, de Araujo Furtado M, Niquet J, Linz EO, Schultz CR, et al. Efficacy of Lacosamide and Rufinamide as Adjuncts to Midazolam-Ketamine Treatment Against Cholinergic-Induced Status Epilepticus in Rats J Pharmacol Exp Ther. 2024 Jan 17;388:347-357.

72. Zhou X, Zeng L, Wang Y, Xu C, Chen Z, Cui S. Discovery of triazenyl triazoles as Na(v)1.1 channel blockers for treatment of epilepsy Bioorg Med Chem Lett. 2022 Nov 1;75:128946.

73. Lin YC, Lai YC, Chou P, Hsueh SW, Lin TH, Huang CS, et al. How Can an Na(+) Channel Inhibitor Ameliorate Seizures in Lennox-Gastaut Syndrome? Ann Neurol. 2021 Jun;89:1099-1113.

74. Metcalf CS, Huff J, Thomson KE, Johnson K, Edwards SF, Wilcox KS. Evaluation of antiseizure drug efficacy and tolerability in the rat lamotrigine-resistant amygdala kindling model Epilepsia Open. 2019 Sep;4:452-463.

75. Gall Z, Orban-Kis K, Szilagyi T. Differential effects of sodium channel blockers on in vitro induced epileptiform activities Arch Pharm Res. 2017 Jan;40:112-121.

76. White HS, Franklin MR, Kupferberg HJ, Schmutz M, Stables JP, Wolf HH. The anticonvulsant profile of rufinamide (CGP 33101) in rodent seizure models Epilepsia. 2008 Jul;49:1213-1220.

77. Masoumi M, Manavi MA, Mohammad Jafari R, Mirzaei A, Hedayatyanfard K, Beigmohammadi MT, et al. Cannabidiol Anticonvulsant Effects Against Lithium-Pilocarpine-Induced Status Epilepticus in Male Rats Are Mediated by Neuroinflammation Modulation and Cannabinoids 1 (CB1), But Not CB2 and GABA(A) Receptors Cannabis Cannabinoid Res. 2024 Jun;9:797-808.

78. Karler R, Turkanis SA. The cannabinoids as potential antiepileptics J Clin Pharmacol. 1981 Aug-Sep;21:437S-448S.

79. Wallace MJ, Wiley JL, Martin BR, DeLorenzo RJ. Assessment of the role of CB1 receptors in cannabinoid anticonvulsant effects Eur J Pharmacol. 2001 Sep 28;428:51-57.

80. Jones NA, Hill AJ, Smith I, Bevan SA, Williams CM, Whalley BJ, et al. Cannabidiol displays antiepileptiform and antiseizure properties in vitro and in vivo J Pharmacol Exp Ther. 2010 Feb;332:569-577.

81. Jones NA, Glyn SE, Akiyama S, Hill TD, Hill AJ, Weston SE, et al. Cannabidiol exerts anti-convulsant effects in animal models of temporal lobe and partial seizures Seizure. 2012 Jun;21:344-352.

82. Do Val-da Silva RA, Peixoto-Santos JE, Kandratavicius L, De Ross JB, Esteves I, De Martinis BS, et al. Protective Effects of Cannabidiol against Seizures and Neuronal Death in a Rat Model of Mesial Temporal Lobe Epilepsy Front Pharmacol. 2017;8:131.

83. Kollipara R, Langille E, Tobin C, French CR. Phytocannabinoids Reduce Seizures in Larval Zebrafish and Affect Endocannabinoid Gene Expression Biomolecules. 2023 Sep 16;13.

84. Rosenberg EC, Chamberland S, Bazelot M, Nebet ER, Wang X, McKenzie S, et al. Cannabidiol modulates excitatory-inhibitory ratio to counter hippocampal hyperactivity Neuron. 2023 Apr 19;111:1282-1300 e1288.

85. Patel DC, Wallis G, Fujinami RS, Wilcox KS, Smith MD. Cannabidiol reduces seizures following CNS infection with Theiler's murine encephalomyelitis virus Epilepsia Open. 2019 Sep;4:431-442.

86. Vilela LR, Lima IV, Kunsch EB, Pinto HPP, de Miranda AS, Vieira ELM, et al. Anticonvulsant effect of cannabidiol in the pentylenetetrazole model: Pharmacological mechanisms, electroencephalographic profile, and brain cytokine levels Epilepsy Behav. 2017 Oct;75:29-35.

87. Dlugosz L, Zhou HZ, Scott BW, Burnham M. The effects of cannabidiol and Delta9-tetrahydrocannabinol, alone and in combination, in the maximal electroshock seizure model Epilepsy Res. 2023 Feb;190:107087.

88. Patra PH, Barker-Haliski M, White HS, Whalley BJ, Glyn S, Sandhu H, et al. Cannabidiol reduces seizures and associated behavioral comorbidities in a range of animal seizure and epilepsy models Epilepsia. 2019 Feb;60:303-314.

89. Socala K, Wyska E, Szafarz M, Nieoczym D, Wlaz P. Acute effect of cannabidiol on the activity of various novel antiepileptic drugs in the maximal electroshock- and 6 Hz-induced seizures in mice: Pharmacodynamic and pharmacokinetic studies Neuropharmacology. 2019 Nov 1;158:107733.

90. Golub V, Ramakrishnan S, Reddy DS. Isobolographic analysis of adjunct antiseizure activity of the FDA-approved cannabidiol with neurosteroids and benzodiazepines in adult refractory focal onset epilepsy Exp Neurol. 2023 Feb;360:114294.

91. Gong X, Liu L, Li X, Xiong J, Xu J, Mao D, et al. Neuroprotection of cannabidiol in epileptic rats: Gut microbiome and metabolome sequencing Front Nutr. 2022;9:1028459.

92. Rana RR, Rajasekaran K, Knappertz V, Gray RA. Pharmacodynamic synergism contributes to the antiseizure action of cannabidiol and clobazam Exp Neurol. 2023 Feb;360:114286.

93. Witherspoon E, Quinlan S, Forcelli PA. Preclinical efficacy of cannabidiol for the treatment of early-life seizures Pharmacol Rep. 2022 Oct;74:1092-1098.

94. Reddy DS, Mbilinyi RH, Ramakrishnan S. Efficacy of the FDA-approved cannabidiol on the development and persistence of temporal lobe epilepsy and complex focal onset seizures Exp Neurol. 2023 Jan;359:114240.

95. Lazarini-Lopes W, Campos-Rodriguez C, Garcia-Cairasco N, N'Gouemo P, Forcelli PA. Cannabidiol attenuates generalized tonic-clonic and suppresses limbic seizures in the genetically epilepsy-prone rats (GEPR-3) strain Pharmacol Rep. 2023 Feb;75:166-176.

96. Roebuck AJ, Greba Q, Onofrychuk TJ, McElroy DL, Sandini TM, Zagzoog A, et al. Dissociable changes in spike and wave discharges following exposure to injected cannabinoids and smoked cannabis in Genetic Absence Epilepsy Rats from Strasbourg Eur J Neurosci. 2022 Feb;55:1063-1078.

97. de Assis Lima IV, Pinto HPP, Bellozi PMQ, da Silva MCM, Vilela LR, Moreira FA, et al. Cannabidiol effect in pentylenetetrazole-induced seizures depends on PI3K Pharmacol Rep. 2022 Oct;74:1099-1106.

98. Gall Z, Kelemen K, Tolokan A, Zolcseak I, Savel I, Bod R, et al. Anticonvulsant Action and Long-Term Effects of Chronic Cannabidiol Treatment in the Rat Pentylenetetrazole-Kindling Model of Epilepsy Biomedicines. 2022 Jul 28;10.

99. Costa AM, Russo F, Senn L, Ibatici D, Cannazza G, Biagini G. Antiseizure Effects of Cannabidiol Leading to Increased Peroxisome Proliferator-Activated Receptor Gamma Levels in the Hippocampal CA3 Subfield of Epileptic Rats Pharmaceuticals (Basel). 2022 Apr 19;15.

100. Janisset N, Romariz SAA, Hashiguchi D, Quintella ML, Gimenes C, Yokoyama T, et al. Partial protective effects of cannabidiol against PTZ-induced acute seizures in female rats during the proestrus-estrus transition Epilepsy Behav. 2022 Apr;129:108615.

101. Uttl L, Hlozek T, Mares P, Palenicek T, Kubova H. Anticonvulsive Effects and Pharmacokinetic Profile of Cannabidiol (CBD) in the Pentylenetetrazol (PTZ) or N-Methyl-D-Aspartate (NMDA) Models of Seizures in Infantile Rats Int J Mol Sci. 2021 Dec 22;23.

102. Lazarini-Lopes W, Do Val-da Silva RA, da Silva-Junior RMP, Silva-Cardoso GK, Leite-Panissi CRA, Leite JP, et al. Chronic cannabidiol (CBD) administration induces anticonvulsant and antiepileptogenic effects in a genetic model of epilepsy Epilepsy Behav. 2021 Jun;119:107962.

103. Frias-Soria CL, Perez-Perez D, Orozco-Suarez S, Rocha L. Cannabidiol modifies the seizure expression and effects of antiseizure drugs in a rat model of recurrent severe seizures Seizure. 2021 Aug;90:67-73.

104. Cabral-Pereira G, Sanchez-Benito D, Diaz-Rodriguez SM, Goncalves J, Sancho C, Castellano O, et al. Behavioral and Molecular Effects Induced by Cannabidiol and Valproate Administration in the GASH/Sal Model of Acute Audiogenic Seizures Front Behav Neurosci. 2020;14:612624.

105. Goerl B, Watkins S, Metcalf C, Smith M, Beenhakker M. Cannabidiolic acid exhibits entourage-like improvements of anticonvulsant activity in an acute rat model of seizures Epilepsy Res. 2021 Jan;169:106525.

106. Yu Y, Yang Z, Jin B, Qin X, Zhu X, Sun J, et al. Cannabidiol inhibits febrile seizure by modulating AMPA receptor kinetics through its interaction with the N-terminal domain of GluA1/GluA2 Pharmacol Res. 2020 Nov;161:105128.

107. Mascal M, Hafezi N, Wang D, Hu Y, Serra G, Dallas ML, et al. Synthetic, non-intoxicating 8,9-dihydrocannabidiol for the mitigation of seizures Sci Rep. 2019 May 23;9:7778.

108. Rodriguez-Munoz M, Onetti Y, Cortes-Montero E, Garzon J, Sanchez-Blazquez P. Cannabidiol enhances morphine antinociception, diminishes NMDA-mediated seizures and reduces stroke damage via the sigma 1 receptor Mol Brain. 2018 Sep 17;11:51.

109. Friedman LK, Wongvravit JP. Anticonvulsant and Neuroprotective Effects of Cannabidiol During the Juvenile Period J Neuropathol Exp Neurol. 2018 Oct 1;77:904-919.

110. Pelz MC, Schoolcraft KD, Larson C, Spring MG, Lopez HH. Assessing the role of serotonergic receptors in cannabidiol's anticonvulsant efficacy Epilepsy Behav. 2017 Aug;73:111-118.

111. Hosseinzadeh M, Nikseresht S, Khodagholi F, Naderi N, Maghsoudi N. Cannabidiol Post-Treatment Alleviates Rat Epileptic-Related Behaviors and Activates Hippocampal Cell Autophagy Pathway Along with Antioxidant Defense in Chronic Phase of Pilocarpine-Induced Seizure J Mol Neurosci. 2016 Apr;58:432-440.

112. Mao K, You C, Lei D, Zhang H. High dosage of cannabidiol (CBD) alleviates pentylenetetrazole-induced epilepsy in rats by exerting an anticonvulsive effect Int J Clin Exp Med. 2015;8:8820-8827.

113. Gobira PH, Vilela LR, Goncalves BD, Santos RP, de Oliveira AC, Vieira LB, et al. Cannabidiol, a Cannabis sativa constituent, inhibits cocaine-induced seizures in mice: Possible role of the mTOR pathway and reduction in glutamate release Neurotoxicology. 2015 Sep;50:116-121.

114. Shirazi-zand Z, Ahmad-Molaei L, Motamedi F, Naderi N. The role of potassium BK channels in anticonvulsant effect of cannabidiol in pentylenetetrazole and maximal electroshock models of seizure in mice Epilepsy Behav. 2013 Jul;28:1-7.

115. Consroe P, Martin A, Singh V. Antiepileptic potential of cannabidiol analogs J Clin Pharmacol. 1981 Aug-Sep;21:428S-436S.

116. Karler R, Borys HK, Turkanis SA. Influence of 22-day treatment on the anticonvulsant properties of cannabinoids Naunyn Schmiedebergs Arch Pharmacol. 1982 Aug;320:105-109.

117. Karler R, Turkanis SA. Subacute cannabinoid treatment: anticonvulsant activity and withdrawal excitability in mice Br J Pharmacol. 1980 Mar;68:479-484.

118. Consroe P, Wolkin A. Cannabidiol--antiepileptic drug comparisons and interactions in experimentally induced seizures in rats J Pharmacol Exp Ther. 1977 Apr;201:26-32.

119. Javadzadeh Y, Santos A, Aquilino MS, Mylvaganam S, Urban K, Carlen PL. Cannabidiol Exerts Anticonvulsant Effects Alone and in Combination with Delta(9)-THC through the 5-HT1A Receptor in the Neocortex of Mice Cells. 2024 Mar 7;13.

120. Wilkinson JD, Whalley BJ, Baker D, Pryce G, Constanti A, Gibbons S, et al. Medicinal cannabis: is delta9-tetrahydrocannabinol necessary for all its effects? J Pharm Pharmacol. 2003 Dec;55:1687-1694.

121. Costa AM, Senn L, Anceschi L, Brighenti V, Pellati F, Biagini G. Antiseizure Effects of Fully Characterized Non-Psychoactive Cannabis sativa L. Extracts in the Repeated 6-Hz Corneal Stimulation Test Pharmaceuticals (Basel). 2021 Dec 3;14.

122. Pertwee RG, Browne SE, Ross TM, Stretton CD. An investigation of the involvement of GABA in certain pharmacological effects of delta-9-tetrahydrocannabinol Pharmacol Biochem Behav. 1991 Nov;40:581-585.

123. Karler R, Cely W, Turkanis SA. Anticonvulsant properties of delta 9-tetrahydrocannabinol and other cannabinoids Life Sci. 1974 Sep 1;15:931-947.

124. Turkanis SA, Smiley KA, Borys HK, Olsen DM, Karler R. An electrophysiological analysis of the anticonvulsant action of cannabidiol on limbic seizures in conscious rats Epilepsia. 1979 Aug;20:351-363.

125. Klein BD, Jacobson CA, Metcalf CS, Smith MD, Wilcox KS, Hampson AJ, et al. Evaluation of Cannabidiol in Animal Seizure Models by the Epilepsy Therapy Screening Program (ETSP) Neurochem Res. 2017 Jul;42:1939-1948.

126. Carlini EA, Mechoulam R, Lander N. Anticonvulsant activity of four oxygenated cannabidiol derivatives Res Commun Chem Pathol Pharmacol. 1975 Sep;12:1-15.

127. Samarut E, Nixon J, Kundap UP, Drapeau P, Ellis LD. Single and Synergistic Effects of Cannabidiol and Delta-9-Tetrahydrocannabinol on Zebrafish Models of Neuro-Hyperactivity Front Pharmacol. 2019;10:226.

128. Thornton C, Dickson KE, Carty DR, Ashpole NM, Willett KL. Cannabis constituents reduce seizure behavior in chemically-induced and scn1a-mutant zebrafish Epilepsy Behav. 2020 Sep;110:107152.

129. Chuang SH, Westenbroek RE, Stella N, Catterall WA. Combined Antiseizure Efficacy of Cannabidiol and Clonazepam in a Conditional Mouse Model of Dravet Syndrome J Exp Neurol. 2021;2:81-85.

130. Jansen NA, Perez C, Schenke M, van Beurden AW, Dehghani A, Voskuyl RA, et al. Impaired theta-gamma Coupling Indicates Inhibitory Dysfunction and Seizure Risk in a Dravet Syndrome Mouse Model J Neurosci. 2021 Jan 20;41:524-537.

131. Anderson LL, Absalom NL, Abelev SV, Low IK, Doohan PT, Martin LJ, et al. Coadministered cannabidiol and clobazam: Preclinical evidence for both pharmacodynamic and pharmacokinetic interactions Epilepsia. 2019 Nov;60:2224-2234.

132. Anderson LL, Low IK, McGregor IS, Arnold JC. Interactions between cannabidiol and Delta(9) -tetrahydrocannabinol in modulating seizure susceptibility and survival in a mouse model of Dravet syndrome Br J Pharmacol. 2020 Sep;177:4261-4274.

133. Kaplan JS, Stella N, Catterall WA, Westenbroek RE. Cannabidiol attenuates seizures and social deficits in a mouse model of Dravet syndrome Proc Natl Acad Sci U S A. 2017 Oct 17;114:11229-11234.

134. Li X, Yennawar M, Wiest A, O'Brien WT, Babrowicz B, White RS, et al. Cannabidiol attenuates seizure susceptibility and behavioural deficits in adult CDKL5(R59X) knock-in mice Eur J Neurosci. 2024 Jun;59:3337-3352.

135. Gu B, Zhu M, Glass MR, Rougie M, Nikolova VD, Moy SS, et al. Cannabidiol attenuates seizures and EEG abnormalities in Angelman syndrome model mice J Clin Invest. 2019 Dec 2;129:5462-5467.

136. Dearborn JT, Nelvagal HR, Rensing NR, Takahashi K, Hughes SM, Wishart TM, et al. Effects of chronic cannabidiol in a mouse model of naturally occurring neuroinflammation, neurodegeneration, and spontaneous seizures Sci Rep. 2022 Jul 4;12:11286.

**Supplementary tables**

| **Tables S1.** List of strings used for the search. | | | |
| --- | --- | --- | --- |
| ID number | Search | Strings |  |
| 1 | Fenfluramine | ("fenfluramin*") OR ("dexfenfluramin*") OR ("levofenfluramin*") |  |
| 2 | Stiripentol | ("stiripentol") OR ("diacomit") OR ("D-306") OR ("BCX" AND "2060") |  |
| 3 | Ganaxolone | "ganaxolone" OR "ztalmy" OR "GANAXOLONE" OR "38398-32-2" OR "CCD 1042" |  |
| 4 | Cannabidiol | "cannabidiol"[MeSH Terms] OR CBD OR Epidiolex |  |
| 5 | Everolimus | "everolimus" OR "RAD" OR "SDZ-RAD" OR "159351-69-6" OR "NSC733504" |  |
| 6 | Rufinamide | “xilep” OR “inovelon” OR “rufinamide” [Supplementary Concept]) |  |
| 7 | Acute seizure models | "acut*" AND "seizure*" AND ("model*" OR "test*") |  |
| 8 | Maximal electroshock seizure / Maximal electroshock seizure threshold | ("mes model") OR ("maximal electroshock" AND "test") OR ("maximal electroshock" AND "seizure*") |  |
| 9 | 6-Hz stimulation | ("6 Hz") AND ("electric*" AND "stimulation") |  |
| 10 | Subcutaneous pentylenetetrazole | ("subcutaneous*") AND ("pentylenetetrazol*") |  |
| 11 | Intravenous pentylenetetrazole | ("intravenous*") AND ("pentylenetetrazol*") |  |
| 12 | Benzodiazepine-resistant status epilepticus | ("benzodiazepine-resistant" OR ("bzd" AND "resistan*") OR "resistan*") AND "status epilepticus" AND "pilocarpine" |  |
| 13 | Chronic seizure models | "chronic*" AND "seizure*" AND ("model*" OR "test*") |  |
| 14 | Corneal kindling | (corneal kindl*) AND ("seizure*" OR "test") |  |
| 15 | Intrahippocampal kainate model | ("hippocampal kain*" OR "intrahippocampal kain*") AND ("model" OR "temporal lobe epilepsy") |  |
| 16 | Intraamygdala kainate model | ("amygdal*" AND "kain*") AND ("model" OR "temporal lobe epilepsy") |  |
| 17 | Lamotrigine-resistant kindling model | ("lamotrigine" AND "resistant") AND "kind*" AND "model" |  |
| 18 | Drug resistant epilepsy models | (("drug*" AND "resistan*" AND "epilep*") AND ("model*" OR "test*")) NOT ("human*" OR "patient*") |  |
| 19 | Post-status epilepticus epilepsy models | ("spontaneous recurrent seizures") AND ("kain*") AND ("chronic" OR "epilepsy") |  |
| 20 | Zebrafish | "zebrafish"[MeSH Terms] OR "zebrafish"[All Fields] OR "zebrafishes"[All Fields] OR "zebrafish s"[All Fields] |  |
| 21 | DEEs | ("epileptic encephalopath*") OR ("development* encephalopathy") |  |
| 22 | Dravet syndrome | "dravet syndrome" OR "epilepsies, myoclonic" OR "myoclonic epilepsy" OR "dravet" |  |
| 23 | Lennox Gastaut syndrome | "lennox gastaut syndrome" OR "lennox-gastaut syndrome" |  |
| 24 | CDKL5 deficiency disorder | "cdkl5 deficiency" OR "cyclin dependent kinase like 5 deficiency" OR "cyclin-dependent kinase-like 5 disorder" OR "cdkl5 deficien*" OR "cyclin dependent kinase like 5 deficien*" |  |
| 25 | Tuberous sclerosis complex | "tuberous sclerosis" OR "tuberous sclerosis complex" |  |
| 26 | Animal models | "models, animal"[MeSH Terms] OR "animal models" OR "preclinic* evaluation" OR "preclinic* models" |  |

| **Table S2**. Literature information: preclinical data for fenfluramine in non-specific/general and specific models of epilepsy and developmental epileptic encephalopathies. | | | | | | |
| --- | --- | --- | --- | --- | --- | --- |
| Type of model | Reference | Species (sex, strain) | Model(s) | Drug exposure (Dose /concentration; route of administration; PTT or exposure time) | Main results | Comments |
| General/non-specific models | ^7^ | Rats (male, CD-COBS) | s.c. PTZ | 5 mg/kg; i.p.; 0.5 h | Reduced incidence of tonic seizures.  Reduced mortality. | D-fenfluramine |
|  | ^8^ | Gerbils (male and female)  Mice (male, NMRI) | Pressure-induced seizures (gerbils)  MES (mice)  MEST (mice) | max. 10 mg/kg; i.p.; 0.5 (gerbils and mice) and 2 h (only gerbils) | No effects on pressure induced seizures or MES. Increased MEST. | No specification for racemic form or enantiomers |
|  | ^9^ | Rats (Wistar)* | Mg^2+^-induced epileptiform activity in rat’s entorhinal cortex | 0.2 – 1 mM; bath; - | Reduced epileptiform activity. | No specification for racemic form or enantiomers |
|  | ^10^ | Mice (male, CD-1)  Rats (male, Sprague-Dawley and Wistar) | MES  MEST  s.c. PTZ  Only in mouse:  6 Hz 32/44 mA  i.v. PTZ | 5 – 20 mg/kg; i.p.; 45 min | No effects in any model, except for an increased MEST in mice and rats. | No specification for racemic form or enantiomers |
|  | ^11^ | Mice (male, CF-1) | MES  6 Hz 44 mA  cKDL | 0.25 – 60 mg/kg; i.p.; 4 h | Antiseizure effects in the MES and 6Hz 44 mA models.  Minimal antiseizure effects in the fully kindled mice. | The effective dose in the 6 Hz 44 mA model also induced motor impairment |
|  | ^12^ | Mice (male, CF-1) | 6 Hz 16/22/44 mA | 1 – 30 mg/kg; i.p.; - | Antiseizure effects of fenfluramine only in the 22mA intensity. | No specification for racemic form or enantiomers.  Fenfluramine’s convulsant effects at 30 mg/kg |
|  | ^13^ | Mice (male and female, DBA/1) | Noise-induced seizures | 5 – 40 mg/kg; i.p.; 0.5 – 24 h | Antiseizure effects for up to 24 h. | Racemic fenfluramine hydrochloride |
|  | ^14^ | Mice (male and female, DBA/1) | Noise-induced seizures | 15 mg/kg; i.p.; 16 h | Antiseizure effects. | Fenfluramine’s effects modulated by 5-HT serotonin receptor modulators |
|  | ^15^ | Zebrafish (male, AB) | i.p. PTZ | 10 mg/kg; i.p.; 10 min | Antiseizure effects. | No specification for racemic form or enantiomers |
|  | ^16^ | Rats (male, Sprague CD IGS Dawley)  Mice (male, CF-1) | MES  Only mice:  6 Hz 44 mA | Rats:  5 – 15 mg/kg; i.p.; 0.25 – 4 h  Mice:  10 – 30 mg/kg; i.p.; 0.5 – 6 h | Antiseizure effects in the MES but not in the 6 Hz 44 mA model. | Racemic fenfluramine hydrochloride |
|  | ^17^ | Mice (male, CD-1; male, DBA/2) | MES  Noise-induced seizures | -; i.p.; 1 h | MES:  ED_50_ = 8.1 mg/kg (peak of effect 6 h after administration; PI = 5.5).  Audiogenic seizures:  ED_50_ = 11.8 mg/kg. | ED_50_ information also for D-fenfluramine and L-fenfluramine. |
|  | ^18^ | Mice (female, NMRI; male, C57BL/6) | aKDL  IHK | 0.03 – 10 mg/kg; i.p.; 2 h | aKDL:  Minimal antiseizure effects in the fully kindled mice.  IHK:  Antiseizure effects for up to 2 h after administration. | Racemic fenfluramine hydrochloride |
|  | ^19^ | Mice (male, CD-1) | NMDA-induced seizures | 3 nM; i.c.v.; 0.5 h | Antiseizure effects. | D-fenfluramine |
| Dravet syndrome | ^20^ | Zebrafish (AB-Scn1a^-/-^) | Spontaneous epileptiform activity | -; bath; 22 h | Antiseizure effects. | No specification for racemic form or enantiomers |
|  | ^21^ | Zebrafish (AB-Scn1a^-/-^) | Spontaneous epileptiform activity  Hyperthermia-induced seizures | 12.5 – 50 μM; bath; 24 h | Spontaneous epileptiform activity: Antiseizure effects.  Hyperthermia-induced seizures: No effects. | No specification for racemic form or enantiomers |
|  | ^22^ | Zebrafish (AB-Scn1a^-/-^) | Spontaneous epileptiform activity | -; bath; 24 h | Antiseizure effects. | Racemic fenfluramine |
|  | ^23^ | Zebrafish (AB-Scn1a^-/-^) | Spontaneous epileptiform activity | 50 μM; bath; 24 h | Antiseizure effects. | Racemic fenfluramine |
|  | ^24^ | Mice (Scn1a^A1783V/+^) | Hyperthermia-induced seizures | 10 and 25 mg/kg; i.p.; 0.5 and 4 h | No antiseizure effects. | No specification for racemic form or enantiomers |
| SCN8A-related epilepsy | ^12^ | Mice (Scn8a^R1620L/+^) | 6 Hz 16 mA | 17 mg/kg; i.p.; - | No antiseizure effects. | No specification for racemic form or enantiomers |
| *, The tissue was obtained from these species/strains.  PTT, pre-treatment time; s.c., subcutaneous; i.p., intraperitoneal; PTZ, Pentylenetetrazole; i.p., intraperitoneal; i.v., intravenous; MES, Maximal electroshock seizure; MEST, MES Threshold; KDL, Kindling; cKDL, corneal KDL; aKDL; amygdala KDL; IHK, Intrahippocampal kainic acid mouse model; NMDA; n-methyl-d-aspartate; i.c.v., intracerebroventricular; ED_50_, median effective dose; PI, protective index (median toxic dose/ ED_50_). | | | | | | |

| **Table S3.** Literature information: preclinical data for stiripentol in non-specific/general and specific models of epilepsy and developmental epileptic encephalopathies. | | | | | | |
| --- | --- | --- | --- | --- | --- | --- |
| Type of model | Reference | Species (sex, strain) | Model(s) | Drug exposure (Dose/concentration; route of administration; PTT or exposure time) | Main results | Comments |
| General/non-specific model | ^25^ | Rats (male, Wistar) | s.c. PTZ  SE | 150 – 350 mg/kg; i.p.; 1 h | Antiseizure effects. | Effects only in young animals. |
|  | ^26^ | Mice (male and female, Kunming) | s.c. PTZ  MES | 100 mg/kg; i.p.; 0.5 – 3 h (MES) and 0.5 h (PTZ) | Antiseizure effects in both models. |  |
|  | ^27^ | Mice (male, Swiss) | MES | 250 – 325 mg/kg; i.p.; 1 h | Antiseizure effects. |  |
|  | ^28^ | Mice (male, Swiss) | s.c. PTZ | -; i.p.; 1 h | ED_50_ = 221.3 mg/kg (PI = 2.89). |  |
|  | ^29^ | Mice (male, Swiss) | MES | 225 – 350 mg/kg; i.p.; 1 h | ED_50_ = 277.7 mg/kg. |  |
|  | ^30^ | Rats (male, Sprague-Dawley) | i.v. PTZ | 150 – 900 mg/kg; i.p.; 1 h | Antiseizure effects. |  |
|  | ^31^ | Mice (ICR) | IHK | 300 mg/kg; i.p.; - | No antiseizure effects. |  |
|  | ^32^ | Rats (male, Sprague-Dawley) | SE | 10 – 1000 mg/kg; -; at onset or 45 min after stage 3 seizures. | Antiseizure effects. | Better effects in young animals. |
|  | ^33^ | Rats (male, Sprague-Dawley) | SE | 10 – 1000 mg/kg; i.p.; at onset or 45 min after stage 3 seizures. | At onset:  ED_50_ = 100 (young) and 377.6 mg/kg (adults).  45 min after stage 3 seizures:  ED_50_ = 216.3 (young) and 397.2 mg/kg (adults). |  |
|  | ^34^ | Mice (male, Swiss-Webster) | Cocaine-induced seizures | -; i.p.; 1 h | ED_50_ = 68.3 mg/kg (PI = 5.3) |  |
|  | ^35^ | Rats (male, Sprague-Dawley)* | In vitro spontaneous epileptiform activity | 100 and 300 μM; bath; 20 min | Antiseizure effects. | Reduction in frequency but increase in duration and amplitude of the epileptiform discharges. |
|  | ^36^ | Mice (male, CBA) | MSO-induced SE | 200-400 mg/kg; i.p.; 0.5 h | Antiseizure effects. |  |
|  | ^37^ | Rats (male, WAG-Rij; male, Sprague-Dawley) | Spontaneous SWD  i.p. PTZ (low dose) | 150-300 mg/kg; i.p.; - | Antiseizure effects in both models. |  |
|  | ^38^ | Rats (male, EOPS)  Mice (EOPS) | MES  i.p. PTZ  Bicuculline  Strychnine | 50-300 mg/kg; i.p.; 0.5 h | MES (rats): ED_50_ = 240 mg/kg  i.p. PTZ (mice): ED_50_ = 200 mg/kg  Antiseizure effects in the other models |  |
| Dravet syndrome | ^39^ | Zebrafish (AB-Scn1a^-/-^) | Spontaneous seizure activity | 6.7 – 667 μM; bath; 10 min | Antiseizure effects. |  |
|  | ^24^ | Mice (Scn1a^A1783V/+^) | Hyperthermia-induced seizures | 100 mg/kg; i.p.; 1 h | No antiseizure effects. |  |
|  | ^40^ | Mice (Scn1a^A1783V/+^) | Spontaneous seizure activity | 150 mg/kg; i.p.; - | No antiseizure effects. |  |
|  | ^23^ | Zebrafish (AB-Scn1a^-/-^) | Spontaneous epileptiform activity | 50 μM; bath; 24 h | No antiseizure effects. |  |
|  | ^41^ | Mice (Scn1a^E1099X/+^) | Hyperthermia-induced seizures | 300 mg/kg; i.p.; 0.5 h | No antiseizure effects. |  |
|  | ^42^ | Mice (Scn1a^R1099X/+^) | Hyperthermia-induced seizures | 300 mg/kg; i.p.; 0.5 h | Antiseizure effects only for 1 month age mice, not for 5 months age mice. | The combination of stiripentol with clobazam was effective in both ages. |
|  | ^43^ | Mice (Gabrg2^Q390X/+^) | Spontaneous seizure activity  i.p. PTZ | 150 mg/kg; i.p.; 1 h | Convulsant effects in both models. |  |
| *, The tissue was obtained from these species/strains.  PTT, pre-treatment time; s.c., subcutaneous; SE, *Status epilepticus;* PTZ, Pentylenetetrazole; i.p., intraperitoneal; MES, Maximal electroshock seizure; i.v., intravenous; IHK, Intrahippocampal kainic acid mouse model; ED_50_, median effective dose; PI, protective index (median toxic dose/ ED_50_); MSO, Methionine sulfoximine; SWD, Spike and wave discharges. | | | | | | |

| Table S4. Literature information: preclinical data for everolimus in non-specific/general and specific models of epilepsy and developmental epileptic encephalopathies. | | | | | | |
| --- | --- | --- | --- | --- | --- | --- |
| Type of model | Reference | Species (sex, strain) | Model(s) | Drug exposure (Dose/concentration; route of administration; PTT or exposure time) | Main results | Comments |
| General/non-specific model | ^44^ | Mice (male, C57BL/6) | IHK | 5 mg/kg; i.p.; qd for 8 weeks | No antiseizure effects |  |
|  | ^45^ | Mice (male, CD-1) | IHK | 5 mg/kg; p.o.; qd for 3 days | No antiseizure effects |  |
|  | ^46^ | Mice (female, NMRI) | MEST | 5 and 10 mg/kg; p.o.; 3 h | Antiseizure effects in both naïve and animals with epilepsy. |  |
|  | ^47^ | Rats (male, CD IGS Sprague Dawley) | Post-SE epilepsy | Starting 1 day after SE:  2 mg/kg; p.o.; qd for 7 days  Starting 1-2 h after SE:  3 mg/kg; p.o.; qd for 5 days | Everolimus did not prevented or modified the development of spontaneous recurrent seizures. |  |
|  | ETSP (ID: 520008) | Mouse (male, C57BL/6) | MES  6 Hz 44 mA  cKDL  IHK | MES, 6Hz 44mA:  3-30 mg/kg; p.o.; 0.5 and 2 h  cKDL:  3-30 mg/kg; p.o.; 1, 2 and 4 h  IHK:  10 and 30 mg/kg; p.o.; - | No antiseizure effects. | There was a reduction of 50% in seizure frequency in the IHK model (n = 3). |
|  | ^48^ | Rats (male, Tsc2^+/-^ and WT Long-Evans) | SE in P11-12 rats. | 1 mg/kg; i.p.; once every 48 h for 12 days | No effects were detected in wet dog shake frequency. | Everolimus reverted behavioral alterations related to the genetic mutation. |
|  | ^49^ | Rats (male, Sprague-Dawley) | Post-SE induced epilepsy | 6 mg/kg; p.o.; 5 days | No antiseizure effects. |  |
| Tuberous sclerosis complex | ^48^ | Rats (male, Tsc2^+/-^ and WT Long-Evans) | SE in P11-12 rats. | 1 mg/kg; i.p.; once every 48 h for 12 days | No effects were detected in wet dog shake frequency. | Everolimus reverted behavioral alterations related to the genetic mutation. |
| Pik3ca associated epilepsy | ^50^ | Mice (Nestin-Cre  Pik3ca^E545K/+^)* | In vitro epileptiform activity | 0.52 μM; bath; 1 h | No antiseizure effects. |  |
| *, The tissue was obtained from these species/strains.  PTT, pre-treatment time; IHK, Intrahippocampal kainic acid mouse model; i.p., intraperitoneal; qd, once per day; p.o., *per os*; MES, Maximal electroshock seizure; MEST, MES Threshold; SE, *Status Epilepticus*; KDL, Kindling; cKDL, corneal KDL. | | | | | | |

| Table S5. Literature information: preclinical data for ganaxolone in non-specific/general and specific models of epilepsy and developmental epileptic encephalopathies. | | | | | | |
| --- | --- | --- | --- | --- | --- | --- |
| Type of model | Reference | Species (sex, strain) | Model(s) | Drug exposure (Dose/concentration; route of administration; PTT or exposure time) | Main results | Comments |
| General/non-specific model | ^51^ | Mouse (male, C57BL/6) | hKDL  6Hz 38 mA | 0.5-10 mg/kg; s.c.; 15 min | 6Hz 38 mA: ED_50_ = 1.46 mg/kg  hKDL: Antiseizure effects in kindled mice. |  |
|  | ^52^ | Mouse (male, Swiss) | SE | 3 mg/kg; i.m.; 40 min after the first myoclonic jerk | Antiseizure effects and reduction in mortality. |  |
|  | ^53^ | Rat (Sprague-Dawley) | Betamethasone-primed rats + NMDA-induced seizures. | Single: 10-50 mg/kg; i.p.; 0.5 h  Repeated: 20 mg/kg; i.p.; bid (P13-P15) | Single and repeating dosing: Antiseizure effects. |  |
|  | ^54^ | Mouse (female, C57BL/6) | aKDL | 1.25-20 mg/kg; s.c.; 15 min | ED_50_ (severity) = 6.6 mg/kg  ED_50_ (ADD): 11 mg/kg |  |
|  | ^55^ | Rat (male, Wistar) | Electricity-induced cortical epileptiform discharges | 5-40 mg/kg; i.p.; after the first AD | Antiseizure effects in rats P25 and P12 but not in P18 rats. | No effects on the motor manifestation of the seizures. |
|  | ^56^ | Rat (male, WAG/Rij) | Spontaneous absence seizures | 0.1-1 nmol/side; i.c. | Antiseizure effects only when injected  in the perioral region of the somatosensory cortex. | Convulsant effects when injected in some areas of the thalamus. |
|  | ^57^ | Mouse (male, Swiss) | 6Hz-32mA | 3-100 mg/kg; i.p.; 15 min | ED_50_ = 6.3mg/kg. |  |
|  | ^58^ | Rat (female, Sprague-Dawley) | s.c. PTZ | 0.625-15 mg/kg; s.c.; 15 min | ED_50_ (naïve) = 3.5 mg/kg (PI = 1.62). | ED_50_ (pseudopregnancy) = 2.38 mg/kg (PI = 3.12)  ED_50_ (catamenial) = 1.2 mg/kg (PI 6.36) |
|  | ^59^ | Rats (male, Sprague-Dawley) | Flurothyl-induced seizures | 0.5-10 mg/kg; i.p.; 10-120 min | Antiseizure effects (dose-dependent). |  |
|  | ^60^ | Lambs (Border Leicester-Merino) | Asphyxia-induced seizures | Bolus: 5 mg/kg; i.v.; 6h after birth.  Repeated: 5 mg/kg/day; i.v.; continuous for 2 days | Antiseizure effects. |  |
|  | ^61^ | Mouse (male and female, C57BL/6; GABA_A_^-/-^) | SE  hKDL  6 Hz 32 mA | hKDL: 1 – 10 mg/kg; s.c.; 15 min  6 Hz 32 mA: 0.6 – 20 mg/kg; s.c.; 15 min | hKDL: Antiseizure effects in kindled animals.  6 Hz 32 mA: ED50 = 1.5 mg/kg (females); 2.9 mg/kg (males). | hKDL: more potent effects in animals with neurosteroid withdrawal syndrome. |
|  | ^62^ | Mouse (male, C57BL/6.129Sv) | s.c. PTZ-KDL | 5.5 mg/kg; i.p.; 15 min | PTZ-KDL: delayed KDL development. |  |
|  | ^34^ | Mouse (male, Swiss) | i.p. PTZ-KDL | 1.38 - 5.43 mg/kg; s.c.; 15 min | Reduction of the frequency of fully kindled mice.  ED_50_ (naïve) = 3.45 mg/kg.  ED_50_ (kindled) = 3.22 mg/kg | No locomotor side effects at these doses. |
|  | ^63^ | Mouse (male, Swiss) | i.p. PTZ-KDL | 0.69-3.45 mg/kg; s.c.; 15 min | Antiseizure effects and delayed the KDL development. | Prevention of other PTZ-induced behaviors. |
|  | ^64^ | Mouse (male, NSA)  Rat (male, Sprague-Dawley) | s.c. PTZ  MES  cKDL  i.v. PTZ threshold  Chemical-induced seizures | -; i.p. and p.o.; - | s.c. PTZ:  ED_50_ (mice, i.p.) = 4.3 mg/kg (PI = 7.8)  ED_50_ (rats, i.p.) = 7.8 mg/kg (PI = 1.8)  ED_50_ (rats, p.o.) = 21 mg/kg (PI = 2.3)  MES  ED_50_ (rats, p.o.) = 58.4 mg/kg (PI = 0.8)  ED_50_ (mice, i.p.) = 29.7 mg/kg (1.1)  cKDL  ED_50_ (rats, i.p.) = 4.5 mg/kg (PI = 3.2)  Antiseizure effects in all the other models. |  |
|  | ^65^ | Rat (Wistar) | s.c. PTZ | 10-60 mg/kg; i.p.; 20 min | Antiseizure effects. | More potency in younger rats |
|  | ^66^ | Rat (female, Sprague-Dawley) | s.c. PTZ | 7 mg/kg; s.c.; bid for 3 or 7 days, 15 min before seizure | ED_50_ = 3.5 mg/kg (PI = 1.6) | No tolerance development. |
|  | ^67^ | Rat (male, Sprague-Dawley) | SE | 6, 9, 12 mg/kg; i.v.; 0, 15, 30, and 60 after the start of SE. | Antiseizure effects and reduced mortality. | Induction of sedative effects. |
| Fragile X syndrome | ^68^ | Mouse (Frm1^-/-^) | Noise-induced seizures | 10 mg/kg; i.p.; 10 min | Antiseizure effects. |  |
| Angelman syndrome | ^69^ | Mouse (Ube3a^tm1Alb^) | Noise-induced seizures  i.p. PTZ | 150 nM; s.c.; continuously for 3 days or 4 weeks (minipump) | Noise-induced seizures: Antiseizure effects (Short- and long-term treatment).  i.p. PTZ: Antiseizure effects (Long-term treatment). |  |
| PTT, pre-treatment time; KDL, Kindling; hKDL, hippocampal KDL; ED_50_, median effective dose; SE, *Status Epilepticus*; i.m., intramuscular; NMDA, n-methyl-d-aspartate; i.p., intraperitoneal; aKDL, amygdala KDL; AD; After-discharge; ADD, AD duration; i.c., intracerebral; ; ED_50_, median effective dose; PI, protective index (median toxic dose/ED_50_); i.v., intravenous; PTZ, Pentylenetetrazole; MES, Maximal electroshock seizure; cKDL; corneal KDL; p.o., *per os;* bid; twice per day; s.c., subcutaneous. | | | | | | |

| Table S6. Literature information: preclinical data for rufinamide in non-specific/general and specific models of epilepsy and developmental epileptic encephalopathies. | | | | | | |
| --- | --- | --- | --- | --- | --- | --- |
| Type of model | Reference | Species (sex, strain) | Model(s) | Drug exposure (Dose/concentration; route of administration; PTT or exposure time) | Main results | Comments |
| General/non-specific model | ^70^ | Mouse (male, BALB/c) | i.p. PTZ-KDL | 50 mg/kg; i.p.; 45-60 min | Prevention of KDL development. | Alone or in combination with Ivermectine and vitamin E.  Protective effects in non-seizure related outcomes (behavior and memory). |
|  | ^71^ | Rat (male, Sprague-Dawley) | SE | 50 mg/kg; i.p.; 40 min after seizure onset. | No significant effects in SE.  Reduced incidence of SRS in treated animals. | Antiseizure effects in the EEG spectral power.  The effects on SE were described in combination with ketamine and midazolam. |
|  | ^72^ | Mouse (male, ICR) | i.p. PTZ  MES | 50 mg/kg; p.o.; 1 h | Antiseizure effects in both models. |  |
|  | ^73^ | Mouse (male, C57BL/6) | AY-9944-induced seizures  i.p. PTZ | Acute AY-9944:  50 mg/kg; s.c.; p3-p6, p8, p14, and p20.  Chronic AY-9944:  50 mg/kg; i.p.; 0.5 h  i.p. PTZ:  50 mg/kg; i.p.; 15 min | AY-9944:  Reduction in SWD in the chronic model.  i.p. PTZ:  Reduction in the frequency of SWD but not from isolated spike bursts. |  |
|  | ^74^ | Rat (male, Sprague-Dawley) | LTG-resistant aKDL | 40 mg/kg; i.p.; 15 min | No antiseizure effects. | All animals presented neurological impairments. |
|  | ^3^ | Rat (male, Sprague-Dawley)  Mouse (male, CF1) | 6Hz 32 and 44 mA  6Hz 60 and 80 V  MES | -; i.p.; 0.5 h | 6Hz 60V (rat):  ED_50_ = 42.7 mg/kg (PI = 8.2)  6Hz 80V (rat):  Antiseizure effects.  MES (rat):  ED_50_ = 7 mg/kg (PI > 50).  6Hz 32 mA (mouse):  ED_50_ = 23.1 mg/kg (PI > 2)  6Hz 44 mA:  ED_50_ = 32.9 mg/kg (PI > 1.4) |  |
|  | ^75^ | Rat (male, Wistar) | 4-AP-induced and low Mg^2+^-induced epileptiform activity (*in vitro*) | -; bath; - | Low-Mg^2+^: Antiseizure effects.  4-AP: antiseizure effects. |  |
|  | ^76^ | Rat (male, Sprague-Dawley)  Mouse (Male, CF1) | MES  s.c. PTZ  acute chemical-induced seizures | MES:  -; i.p.; 15 min (mouse) or 60 min (rat)  -; p.o.; 0.5 h (rat)  Chronic treatment + MES (rat):  6; p.o.; 5-7 days | Mice, i.p.:  ED_50_ (MES) = 15.5 mg/kg (PI > 32.2)  ED50 (s.c. PTZ) = 54 mg/kg (PI > 9.3)  Mice, p.o.:  ED_50_ (MES) = 23.9 mg/kg (PI > 41.9)  ED_50_ (s.c. PTZ): 45.8 mg/kg (PI > 21.9)  Rats, p.o.:  ED_50_ (MES) = 6.1 mg/kg (PI ≈ 163)  No effects in the s.c. PTZ model | No effects on the Strychnine model.  Same effects with chronic and acute dosing. |
| Dravet syndrome | ^24^ | Mouse (Scn1a^A1783/+^) | Hyperthermia-induced seizures | 32 mg/kg; i.p.; 0.5 h | No antiseizure effects. |  |
| PTT, pre-treatment time; PTZ, Pentylenetetrazole; KDL, Kindling; i.p., intraperitoneal; SE, *Status Epilepticus*; SRS, Spontaneous Recurrent Seizures; EEG, Electroencephalogram; MES, Maximal electroshock seizure; p.o., *per os;* s.c., subcutaneous; SWD, Spike-and-Wave Discharges; LTG, Lamotrigine; aKDL, amygdala KDL; ED_50_, median effective dose; PI, protective index (median toxic dose/ ED_50_); 4-AP, 4-Aminopyridine. | | | | | | |

| Table S7. Literature information: preclinical data for cannabidiol in non-specific/general and specific models of epilepsy and developmental epileptic encephalopathies. | | | | | | |
| --- | --- | --- | --- | --- | --- | --- |
| Type of model | Reference | Species (sex, strain) | Model(s) | Drug exposure (Dose/concentration; route of administration; PTT or exposure time) | Main results | Comments |
| General/non-specific model | ^77^ | Rats (male, Wistar) | SE | 1-25 mg/kg; i.p.; 30 min | Antiseizure effects |  |
|  | ^78^ | Mice (male, ICR)  Rats (Sprague-Dawley) | MES  MEST  6 and 60 Hz  s.c. PTZ  Cobalt-induced epilepsy in rats | -; i.p.; - | MES:  ED50 (Mice) = 120 mg/kg (PI = 1.5)  ED50 (Rats) = 50 mg/kg  MES, MEST, and PTZ (mice) = antiseizure effects | No definition of the current used for the 6 and 60 Hz tests |
|  | ^79^ | Mice (male, CF-1) | MES | -; i.p.; 2 h | ED_50_ = 80 mg/kg |  |
|  | ^80^ | Rats (male and female, Wistar Kyoto) | Mg^2+^-Free and 4-AP in vitro induced epileptiform activity.  i.p. PTZ | In vitro:  0.01-100 μM; bath; 30 min  In vivo:  1, 10, 100 mg/kg; i.p.; 1 h | In vitro: antiseizure effects  In vivo: antiseizure effects (only male rats) |  |
|  | ^81^ | Rats (male, Wistar) | Pilocarpine-induced seizures  Penicillin-induced seizures | 1, 10, 100 mg/kg; i.p.; 1 h | Antiseizure effects | Penicillin was administered via i.c.v. |
|  | ^82^ | Rats (male, Wistar) | SE | 10 mg/kg; i.p.; 1 h | Antiseizure effects |  |
|  | ^83^ | Zebrafish (AB) | PTZ | 1-4 uM; medium; 0.5 h | Antiseizure effects |  |
|  | ^84^ | Mice (male, C57BL/6J; GPR55^-/-^)  Rats (male, Wistar Kyoto) | i.p. PTZ  s.c. KA  Post-SE epilepsy | 50, 100, 200 mg/kg; i.p.; 1 h  Post-SE epilepsy:  200 mg/kg; p.o.; continuous (drinking solution) | i.p. PTZ:  Antiseizure effects in wildtype mice  s.c. KA:  Prevention of the increased susceptibility in KA-primed animals | i.p. PTZ:  Antiseizure effects in the EEG |
|  | ^85^ | Mice (male, C57BL/6J) | Theiler’s encephalomyelitis virus  6Hz 32mA | TEMV  150, 180 mg/kg; i.p.; bid from 2 days before to 10 after infection or from 3 to 7 days after infection.  6Hz model  50 mg/kg, i.p.; - | Antiseizure effects in both models (Time of peak effect = 2 h) |  |
|  | ^86^ | Mice (male, Swiss) | i.p. PTZ  s.c. PTZ  i.v. PTZ  i.p. PTZ KDL | 30, 60, 90 mg/kg; i.p.; 0.5 h | Antiseizure effects in the acute models  No effects in KDL progression |  |
|  | ^87^ | Mice (male, CF-1) | MES | 0 – 640 mg/kg; i.p.; 2 h | ED_50_ = 190 mg/kg (PI = 2.4) | The combination with THC showed better effects |
|  | ^88^ | Mice (male, CF-1)  Rats (male, Sprague-Dawley and male, Wistar) | MES  s.c. PTZ  6Hz 32mA  6Hz 44mA  cKDL  SE  Post-SE epilepsy | -; i.p.; 1 h for MES, s.c. PTZ, 6Hz models, and cKDL.  10 mg/kg; i.v.; 1 h for SE  200 mg/kg; p.o.; drinking solution for 8 weeks | Mice ED_50_:  MES = 80 mg/kg (PI = 3.4)  s.c. PTZ = 120 mg/kg (PI = 2.3)  6Hz 32 mA = 144 mg/kg (PI = 1.9)  6Hz 44 mA = 173 mg/kg (PI = 1.6)  cKDL = 115 mg/kg (PI = 2.4)  Rat:  ED_50_ (MES) = 53.2 mg/kg (PI = 9.4)  SE = antiseizure effects  Post-SE epilepsy = antiseizure effects | Cannabidiol also impacted the comorbities in the animals with epilepsy |
|  | ^89^ | Mice (male, Swiss) | MEST  6Hz 32mA | 25, 50, 100 mg/kg; i.p.; 1 h | Antiseizure effects | Increased spontaneous locomotor activity |
|  | ^90^ | Mice (C57BL/6) | 6Hz 38mA | 25-400 mg/kg; i.p.; 1 h | ED_50_ = 53.6 mg/kg | No neurotoxic effects identified  Synergistic effects in combination with ganaxolone |
|  | ^91^ | Rats (male, Sprague Dawley) | Post-SE epilepsy | 20 and 100 mg/kg; p.o.; 0.5 h before SE and for 7 days | SE: Antiseizure effects |  |
|  | ^92^ | Mice (C57BL/6; Swiss/CD1) | MES  6Hz 44 mA | 10-150 mg/kg; i.p.; 1 h | MES:  ED_50_ = 41.9 mg/kg  6Hz 44 mA:  ED_50_ = 49.6 mg/kg |  |
|  | ^93^ | Rats (female, Sprague-Dawley) | s.c. PTZ  MES | 1-200 mg/kg; i.p.; 2 h | s.c. PTZ:  Antiseizure effects, with better response in adolescent animals  MES:  Antiseizure effects |  |
|  | ^94^ | Mice (male, C57Bl/6) | hKDL | KDL progression:  25 and 100 mg/kg; i.p.; qd 1 h after KDL for 14 days  Fully KDL:  25-400 mg/kg; i.p. or p.o.; 1-4 h  KDL retention:  100 mg/kg; i.p.; bid for 14 days | KDL progression: Delayed KDL development  KDL retention:  Reduced seizure burden but no evidence for revert of KDL  KDL fully:  ED_50_ = 52 (i.p.) and 50 (p.o.) mg/kg | U-shaped dose-dependent antiseizure effect  No motor or functional impairments (25-400 mg/kg) in fully kindled animals |
|  | ^95^ | Rats (GEPR-3) | Noise-induced seizures  Noise-induced KDL | 1-100 mg/kg; i.p.; 2 - 6 h | Antiseizure effects |  |
|  | ^96^ | Rats (male, GAERS) | Spontaneous SWD | 10, 30, 100 mg/kg; i.p.; - | Antiseizure effects |  |
|  | ^97^ | Mice (male, C57BL/6; PI3K^-/-^) | i.p. PTZ | 30 mg/kg; i.p.; 0.5 h | Antiseizure effects | The antiseizure were not present in the mutant strain |
|  | ^98^ | Rats (male, Wistar) | i.p. PTZ KDL (progression, fully kindled) | 60 mg/kg; p.o.; 1 h after each PTZ or 24 h before the next injection | PTZ KDL progression:  No evidence for prevention of KDL development  PTZ KDL:  Increased latency to generalized seizures |  |
|  | ^99^ | Rats (male, Sprague-Dawley) | Post-SE epilepsy | 12 or 120 mg/kg; s.c.; bid for 3 days | Antiseizure effects |  |
|  | ^100^ | Rats (female, Sprague-Dawley) | i.p. PTZ | 50 mg/kg; i.p.; 1 h | Antiseizure effects |  |
|  | ^101^ | Rats (male, Wistar) | s.c. PTZ  NMDA-induced seizures | 10 and 60 mg/kg; i.p.; 1 h | s.c. PTZ: Antiseizure effects.  NMDA-induced seizures: no antiseizure effects. | No motor side effects identified |
|  | ^102^ | Rats (male, WAR; Wistar) | Noise-induced KDL (progression, fully kindled) | 25 mg/kg; i.p.; 1 h before seizure bid | Fully kindled:  Antiseizure effects  KDL development: prevention of KDL development | No motor effects identified |
|  | ^103^ | Rats (male, Wistar) | 3MPA-induced seizures | Acute: 200 mg/kg; p.o.; 2 h  Sub-chronic: 50 mg/kg; p.o.; 2 h bid | Acute and sub-chronic administration antiseizure effects | Increased effects in combination with phenobarbital |
|  | ^104^ | Hamster (male, GASH/Sal) | Noise-induced seizures | Acute: 100 mg/kg; i.p.; 45 min  Chronic: 100 mg/kg; i.p.; bid for 2 weeks | No antiseizure effects | Locomotor activity was reduced |
|  | ^105^ | Rats (male, Sprague Dawley) | MES | -; i.p.; 2 h | ED_50_ = 68.8 mg/kg |  |
|  | ^106^ | Mice (C57BL/6) | Hyperthermia- induced seizures in LPS-primed mice. | 3, 10, and 30 mg/kg; i.p.; 1 h | Antiseizure effects |  |
|  | ^107^ | Rats (male; Wistar Han) | i.p. PTZ | 200 mg/kg; i.p.; 1 h | Antiseizure effects |  |
|  | ^108^ | Mice (male, CD1; Sigma1^-/-^) | NMDA-induced seizures | 3 nmol; i.c.v.; 0.5 h | Antiseizure effects | Antiseizure effects absent in the mutant mice |
|  | ^109^ | Rats (male; Sprague Dawley) | i.h. and i.p. KA-induced seizures | 2.18 uM; i.h.; at the same time or 20 min of the KA  10 mg/kg; i.p.; at the same time or after the start of behavioral symptoms | Antiseizure effects | Prevention of KA- induced changes in the open field |
|  | ^110^ | Rats (male, Wistar Kyoto) | i.p. PTZ | 100 mg/kg; i.p.; 1 h | Antiseizure effects | Reduction on mortality |
|  | ^111^ | Rats (Wistar) | Post-SE induced epilepsy | Early post-SE: 100 ng; i.c.v.; for 5 days starting at day 1 post SE Late post-SE: 100 ng; i.c.v.; single dose at 4 weeks after SE | Early:  Delay of the occurrence of the first SRS  Antiseizure effects in both treatment schedules. |  |
|  | ^112^ | Rats (male, Sprague-Dawley) | i.p. PTZ KDL | 10, 20, and 50 mg/kg; i.p.; 1 h | Prevention of KDL development |  |
|  | ^113^ | Mice (male, Swiss) | Cocaine-induced seizures | 15 – 90 mg/kg; i.p.; 0.5 h | Antiseizure effects |  |
|  | ^114^ | Mice (male, NMRI) | i.v. PTZ  MES | i.v. PTZ:  0.2 - 200 ng/mouse; i.c.v.; 10 min  MES:  20, 100, and 200 ng/mouse; i.c.v.; - | i.v. PTZ:  Antiseizure effects MES:  ED_50_ = 26 ng/mouse |  |
|  | ^115^ | Rats (male, AGS) | Noise-induced seizures | -; i.p. and i.v.; - | ED_50_ (i.p., PTT: 2h) = 82.4 mg/kg (PI = 5.5) ED_50_ (i.v., PTT: 0.25h) = 14.9 mg/kg (PI = 2.1) |  |
|  | ^116^ | Mice (male, CD1) | MES  MEST 6 Hz Threshold 60 Hz Threshold | -; i.p., qd for 22 days (1 h on test days) | MES:  ED_50_ = 120 mg/kg  MEST, 6Hz, and 60Hz: Antiseizure effects | Repetitive administration did not modify sensitivity to the 6Hz and 60 Hz models, but it reduce it in the MEST |
|  | ^117^ | Mice (ICR) | MEST 6 Hz Threshold 60 Hz Threshold | 120 mg/kg; qd for 3-4 days (1 h on test days) | Antiseizure effects | Repetitive administration increased the efficacy in the 6Hz model, but not in the MEST and 60 Hz models |
|  | ^118^ | Rats (male, AGS) | MES Noise-induced seizures | -; p.o.; 1 h | MES: ED_50_ = 12 mg/kg (PI = 8.3)  Noise-induced seizure: ED_50_ = 17 mg/kg (PI = 5.9) |  |
|  | ^119^ | Mice (C57BL/6) | In vitro 4-AP-induced epileptiform activity | 10, 30, and 100 μM; bath; 45 min | Antiseizure effects |  |
|  | ^120^ | Rat (-, -) | In vitro oxotremorine-M-induced epileptiform activity | 1 μM; bath; - | No antiseizure effects |  |
|  | ^121^ | Mice (male, CD1) | 6Hz 44 mA | 25 mg/kg; p.o.; qd for 5 days (1 h) | No antiseizure effects |  |
|  | ^122^ | Mice (male, MFI) | i.v. PTZ | 300 mg/kg; -; - | No antiseizure effects |  |
|  | ^123^ | Mice (male, ICR)  Rats (male, Sprague-Dawley) | MES | -; i.p., 1 h | ED_50_ = 118 mg/kg (PI = 1.5) |  |
|  | ^124^ | Rats (male, Sprague-Dawley) | Hippocampal KDL | 0.3-3 mg/kg; i.p.; - | Antiseizure effects |  |
|  | ^125^ | Mice (male, CF-1)  Rats (male, Sprague-Dawley) | MES  6Hz 44 mA  s.c. PTZ  cKDL  LTG-resistant KDL | -; i.p.; -  LTG-resistant KDL: 100-300 mg/kg; i.p.; 2 h | MES:  ED_50_ (mouse, PTT: 2h) = 83.5 mg/kg (PI = 5.1)  ED_50_ (rat, PTT: 2h) = 88.9 mg/kg (PI = 5.6)  6Hz 44mA:  ED_50_ (mouse, PTT: 2h) = 164 mg/kg (PI = 2.6)  s.c. PTZ:  ED_50_ (mouse, PTT: 2h) = 159 mg/kg (PI = 2.7)  cKDL:  ED_50_ (mouse, PTT: 2h) = 119 mg/kg (PI = 3.6)  LTG-resistant KDL: No effects |  |
|  | ^126^ | Mice (male, albino) | MES | 50-200 mg/kg; i.p.; 1-24 h | Antiseizure effects (100 and 200 mg/kg) peak at 1-2 h |  |
|  | ^127^ | Zebrafish (AB) | PTZ | 1 - 2.5 μM; bath; 1 h | Dose-dependent antiseizure effects | Synergic effect in combination with THC |
|  | ^128^ | Zebrafish (AB) | PTZ | 0.3-1 μM; bath; 24 h | Antiseizure effects |  |
|  | ^120^ | Rat | oxotremorine-M-induced epileptiform activity | 1 μM; bath; - | No antiseizure effects |  |
| Dravet syndrome | ^129^ | Mice (SCN1A^+/-^, del of exon 25) | Hyperthermia-induced seizures | 100 mg/kg; i.p.; 1 h | Antiseizure effects | The test was performed only in combination with Clobazam |
|  | ^130^ | Mice (SCN1A^+/-^, del of exon 8) | Spontaneous seizures | 100 mg/kg; i.p.; qd for two days |  | Prevented the progression of the cortical theta-gamma coupling |
|  | ^128^ | Zebrafish (scn1aLab^-/-^) | Spontaneous hyperactivity | 0.3-1 μM; bath; 24 h | Antiseizure effects |  |
|  | ^131^ | Mice (SCN1A^+/-^) | Hyperthermia-induced seizures  Spontaneous seizures | Hyperthermia-induced seizures:  12 and 100 mg/kg; i.p.; 0.5 h  Spontaneous seizures:  3500 mg/kg; p.o.; continuous (chow) from P19 to P30. | No antiseizure effects | The combination with Clobazam displayed antiseizure effects |
|  | ^24^ | Mice (Scn1a^A1783V/+^) | Hyperthermia-induced seizures | 100 and 200 mg/kg; i.p.; 1 h | No antiseizure effects |  |
|  | ^132^ | Mice (Scn1a^tm1Kea^) | Hyperthermia-induced seizures  Spontaneous seizures | Hyperthermia-induced: 12-100 mg/kg; i.p.; 1 h  Spontaneous: 500 and 1000 mg/kg; p.o.; continuous (chow) from P19 to P30 | Hyperthermia-induced seizures: antiseizure effects  Spontaneous seizures: no antiseizure effects | The spontaneous seizures were evaluated in animals primed with a hyperthermia-induced seizure |
|  | ^133^ | Mice (Scn1a^+/-^, del of exon 26) | Spontaneous seizures  Hyperthermia-induced seizures | Hyperthermia induced seizures: 10, 20, 50, 100, 200 mg/kg; i.p.; 1 h  Spontaneous seizures: 100 mg/kg; i.p.; bid from P21 to P27 | Hyperthermia induced seizures: antiseizure effects (100 and 200 mg/kg)  Spontaneous seizures: antiseizure effects | Cannabidiol also positively affected behavioral comorbidities in the Dravet mice |
|  | ^23^ | Zebrafish (ABScn1a^-/-^) | Spontaneous epileptiform activity | 6.25 μM; well; 24 h | No antiseizure effects |  |
| CDKL5-deficiency disorder | ^134^ | Mice (male, CDKL5^R59X/+^) | i.p. PTZ | 100 mg/kg; i.p.; 1 h | Antiseizure effects | Beneficial non-seizure-related effects (memory and learning). |
| GABRA-associated seizures | ^127^ | Zebrafish (gabra1^-/-^) | Light-induced seizures | 1 - 2.5 μM; bath; 1 h | No antiseizure effects | Synergic effect in combination with THC |
| Angelman syndrome | ^135^ | Mice (Ube3a^m+/p-^) | Noise-induced seizures  Flurothyl-KDL  Hyperthermia-induced seizures in KDL mice | Noise-induced: 10, 20, 50, 100 mg/kg; i.p.; 1 h  KDL (acute and hyperthermia): 100 mg/kg; i.p.; 1 h  KDL (chronic): 100 mg/kg; i.p.; qd for 2 weeks. | Noise-induced: dose-dependent antiseizure effects  Acute and chronic KDL: no antiseizure effects  Hyperthermia-induced in KDL mice: antiseizure effects | Cannabidiol displayed a dose-dependent sedative effect more evident in the mutant mice |
| CLN1 disease | ^136^ | Mice (Cln^-/-^) | Spontaneous seizures | 100 mg/kg; p.o.; continuous (gelatin) for 6 months | No antiseizure effects |  |
| SCN8A DEE |  | Mice (Scn8a^R1620L/+^) | 6Hz 16 mA  6Hz 32 mA  s.c. PTZ | 200, 280, 320, 360 mg/kg; i.p.; 2 h | Antiseizure effects |  |
| SE, *Status epilepticus;* i.p., intraperitoneal; PTZ, Pentylenetetrazole; s.c., subcutaneous; KA, Kainic acid; p.o., *per os;* EEG, Electroencephalogram; MES, Maximal electroshock seizure; ED_50_, median effective dose; THC, Tetrahydrocannabinol; KDL, Kindling; hKDL, hippocampal KDL; qd, once per day; bid, twice per day; NMDA, n-methyl-d-aspartate; 3MPA, 3-mercaptopropionic acid; LPS, Lipopolysaccharide; i.c.v., intracerebroventricular; i.h., intrahippocampal; SRS, Spontaneous Recurrent Seizures; i.v., intravenous; PTT, pre-treatment time; MEST, MES Threshold; 4-AP, 4-Aminopyridine; PI, protective index (median toxic dose/ED_50_); cKDL, corneal KDL; LTG, Lamotrigine; TEMV, Theiler’s encephalitis murine virus; SWD, Spike-and-wave discharges. | | | | | | |
